# DNA‐Induced Entropic Gain Triggers an Allosteric Switch for Biomolecular Condensation of Heat Shock Transcription Factor 1

**DOI:** 10.1002/anie.7340537

**Published:** 2026-05-19

**Authors:** Soichiro Kawagoe, Hiroyuki Kumeta, Tomohide Saio

**Affiliations:** ^1^ Institute of Advanced Medical Sciences Tokushima University Tokushima Japan; ^2^ Institute of Photonics and Human Health Frontier Tokushima University Tokushima Japan; ^3^ Faculty of Advanced Life Science Hokkaido University Hokkaido Japan; ^4^ Fujii Memorial Institute of Medical Sciences Tokushima University Tokushima Japan

**Keywords:** allostery, biological phase separation, conformational dynamics, solution NMR, transcription factor

## Abstract

The molecular logic of how site‐specific DNA recognition by a transcription factor (TF) is transduced into macroscopic protein condensation remains a fundamental puzzle in chemical biology. Here, we unveil that the structured DNA‐binding domain (DBD) of a TF acts as an entropic switch to regulate the release of the intrinsically disordered region (IDR). Using high‐resolution solution NMR spectroscopy, we demonstrate that DNA binding significantly shifts the conformational equilibrium of the DBD toward a highly dynamic state. This conformational shift allosterically triggers the release of the IDR, thereby promoting macroscopic biomolecular condensation via multivalent interactions between the IDR and other molecules. Our findings define a mechanism of entropy‐driven allostery, providing a structural and thermodynamic basis for how DNA‐encoded information is transduced into macroscopic phase behavior.

## Graphical abstract

Hsf1 undergoes phase separation around target DNA, activating stress‐responsive transcription. DNA binding tunes conformational dynamics of Hsf1^DBD^, which propagates through large‐scale domain rearrangements involving DBD–IDR interactions to regulate phase separation. This hierarchical framework illustrates how local structural events at the side chain level can be transmitted across multiple spatial scales, leading macroscopic phase changes.

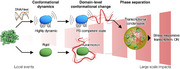

## 1 Introduction

Precise transcriptional regulation is essential for maintaining cellular identity and homeostasis [1]. This regulation requires transcription to be turned on and off with temporal and spatial precision [2]. To this end, transcription factors utilize multifaceted interactions through their DBDs and IDRs [3, 4]. Transcription factors are anchored to specific DNA loci through tight and specific DBD–DNA interactions, and IDRs mediate multivalent interactions that promote the recruitment of other transcription‐related factors through biological phase separation (PS) [4, 5, 6, 7, 8]. However, the spatial regulation mechanism of transcription hubs remains to be fully elucidated.

**FIGURE 1:**
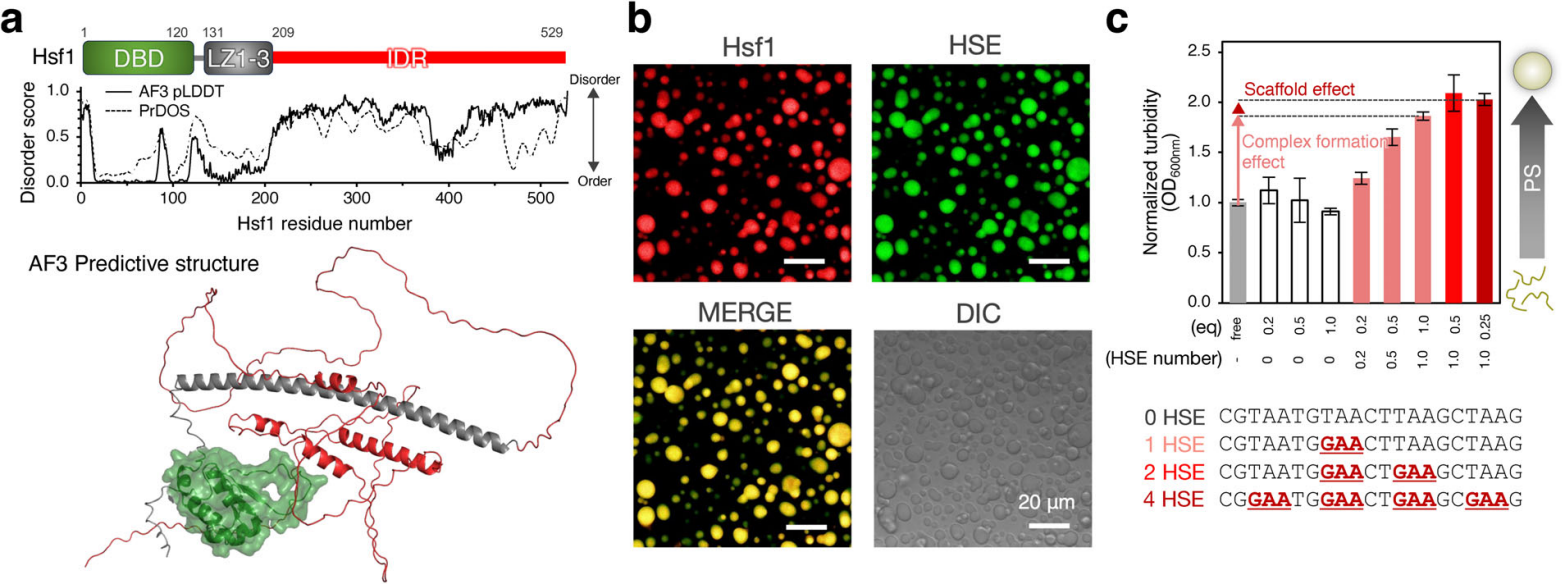
Promotion of Hsf1 phase separation by DNA binding. (a) Domain organization and AlphaFold3 (AF3) [30]‐predicted structure of Hsf1. The plot shows disorder predictions as a function of residue number, based on PrDOS [31] (dashed line), AF3 normalized pLDDT (solid line). DBD, DNA‐binding domain; IDR, intrinsically disordered region; LZ1–3, leucine zipper domains 1–3. (b) Fluorescence and differential interference contrast (DIC) images of Hsf1 (with 0.1 equiv Hsf1–mCherry) and 1 repeat HSE DNA (with 0.1 equiv 6‐FAM‐DNA). The images were acquired at 20 min after induction of PS by reduced pH to 4.5. (c) Turbidity of Hsf1^DBD–IDR^ in the absence (free) and presence of 0, 1, 2, and 4 repeat HSE DNA. Error bars represent the standard deviation from three independent measurements. The turbidity was measured at 5 min after induction of PS by reduced pH to 4.5. PS, phase separation.

Heat shock transcription factor 1 (Hsf1) is a master regulator of genes encoding molecular chaperones and other cytoprotective genes that mediate heat shock response (Figure 1a) [9, 10, 11]. Hsf1 is a stress‐responsive transcription factor that plays a critical role in maintaining proteostasis. The tight control of Hsf1 activity is crucial for cellular homeostasis, as its over‐activation can promote stress tolerance in cancer cells exposed to stresses such as acidification and hypoxia, whereas its insufficient activity is observed in neurodegenerative diseases [12, 13, 14, 15, 16]. In stress response, Hsf1 uses its DBD to recognize a DNA motif called Heat Shock Element (HSE) [17, 18], oligomerizes via leucine zipper regions (LZ1–3) [19, 20, 21, 22, 23], and undergoes PS through multivalent interaction between IDRs, recruiting other transcription factors [24, 25, 26, 27, 28]. Hsf1 condensates are formed at specific loci containing HSEs, suggesting that DNA binding triggers PS and thereby transcriptional activation. A conventional model of PS initiation by the scaffolding effect of DNA is challenged by the fact that Hsf1 condensation occurs at loci containing highly variable numbers of HSE repeats. Although Hsf1 condensation is observed at satellite III DNA containing hundreds of HSEs, which agrees with the scaffolding model, the majority of HSEs are seen in regions containing only 3–9 repeats [19]. Indeed, recent studies have shown that even short HSE sequences are sufficient to promote Hsf1 PS [29], suggesting that DNA binding itself triggers PS of Hsf1. Despite this, the molecular details of how local DNA binding at the DBD is transmitted to the spatially distant IDR to regulate PS remain unclear.

Here, we aimed to elucidate the molecular mechanism by which DNA binding promotes PS of Hsf1. Using solution NMR and biophysical methods, we discovered that modulation of DBD dynamics allosterically regulates IDR assembly and PS. Specifically, our data show that free Hsf1 exists in an autoinhibited state in which IDR is captured by DBD and accordingly protected from intermolecular interaction, whereas DNA binding to DBD increases the conformational dynamics of DBD, leading to the release of IDR from DBD. Importantly, this allosteric change through dynamic modulation is distinct from a simple steric competition between DNA and IDR for DBD which has been often reported in other transcription factors [6, 32, 33, 34, 35, 36, 37, 38]. This unique interplay between structured and disordered domains through entropic modulation can be a new regulatory mechanism for transcription factors.

## 2 Results and Discussions

### 2.1 HSE DNA Promotes the Phase Separation of Hsf1

Because the formation of transcriptional hubs via PS around DNA is a key to transcriptional activation, we first examined the effect of HSE DNA (the nGAAn motif) on Hsf1 PS. Microscopic analysis demonstrated that Hsf1 alone formed droplets in the presence of a crowding agent or under acidic conditions (Figure S1a), and that HSE DNA dissolved into droplets of Hsf1 (Figure 1b). To focus on the effect of DNA on DBD and IDR, we designed an Hsf1^DBD–IDR^ construct lacking LZ1–3. We confirmed that Hsf1^DBD–IDR^ formed droplets with HSE DNA in essentially the same way as did full‐length Hsf1 (Figure S1b). Next, we evaluated the effect of DNA containing varying number of HSE motifs on PS using turbidity assays with Hsf1^DBD‐IDR^. The addition of 0 HSE (nonspecific) DNA had little effect on turbidity, whereas DNA containing a single HSE motif (1 HSE) increased turbidity in a concentration‐dependent manner (Figure 1c). Interestingly, when we compared the conditions containing 1 equiv of 1 HSE, 0.5 equiv of 2 HSE, and 0.25 equiv of 4 HSE—where the total number of HSE motifs was kept constant—we observed only a modest enhancement when multiple HSE motifs were clustered within a single DNA molecule (Figures 1c and S1c). These results suggest that Hsf1‐DNA complex formation itself triggers PS through an unidentified mechanism.

### 2.2 DNA Binding Disrupts the DBD–IDR Interdomain Interactions of Hsf1

**FIGURE 2:**
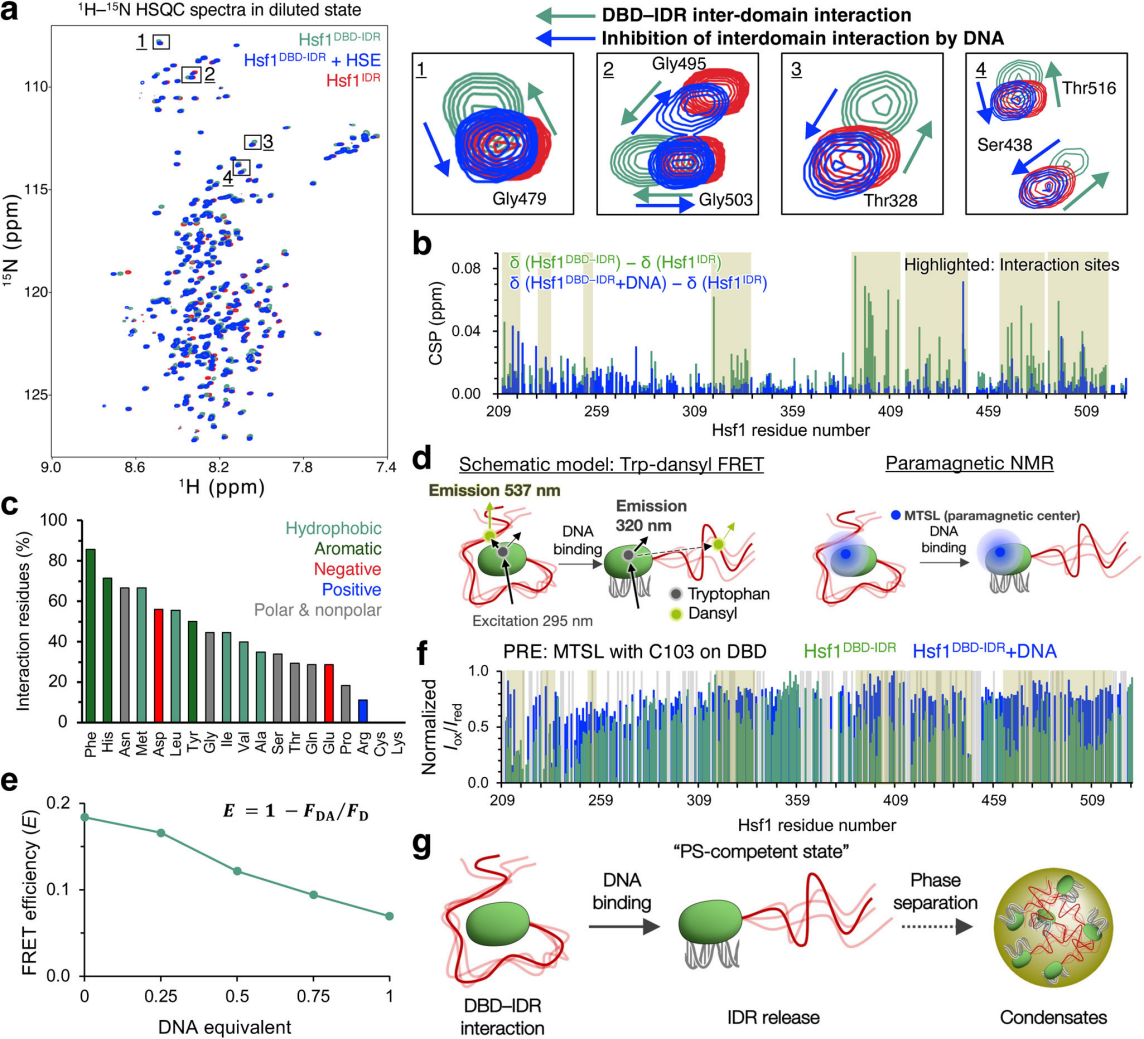
The conformational rearrangements of Hsf1 upon DNA binding. (a) ^1^H–^15^N HSQC spectra of Hsf1^IDR^ (red), free Hsf1^DBD–IDR^ (green), and Hsf1^DBD‐IDR^ in complex with unlabeled HSE DNA (blue). Selected regions (boxes 1–4) are shown as expanded views, highlighting representative IDR resonances that exhibit CSDs by interdomain interaction with the DBD (green arrows) and their reductions by DNA binding (blue arrows). (b) CSDs between Hsf1^IDR^ and Hsf1^DBD–IDR^ in the absence (green) and presence (blue) of HSE DNA, plotted as a function of residue number. The yellow‐shaded regions highlight segments showing pronounced CSDs and accordingly defined as DBD‐binding sites. (c) Amino acid composition of the DBD‐binding sites in IDR normalized against the total number of each amino acid in the IDR sequence. (d) Schematic representation of Trp–dansyl FRET and PRE experiments. Hsf1^DBD–IDR^ contains two tryptophan residues, Trp23 and Trp37, both located in DBD. In the FRET experiments, all endogenous cysteine residues in Hsf1^DBD–IDR^ were substituted with serine, and Ser485 within IDR was mutated to cysteine for site‐specific labeling with dansyl‐maleimide. In the PRE experiments, all cysteine residues except Cys103 were substituted with serine, and MTSL was introduced at the remaining Cys103 on DBD. (e) Trp–dansyl FRET experiment. The decrease in Trp–dansyl FRET efficiency upon DNA addition indicates that DNA binding causes dissociation of DBD and IDR. (f) Per‐residue PRE intensity ratio (*I*_ox_/*I*_red_) for Hsf1^DBD‐IDR^ C103‐MTSL in the absence (green) and presence of 2 equiv HSE DNA (blue). Gray bars indicate unassigned signals. (g) Proposed model for DNA binding‐mediated promotion of Hsf1 PS. DNA inhibits DBD–IDR interdomain interaction and promotes PS by facilitating intermolecular interactions between IDRs.

**FIGURE 3:**
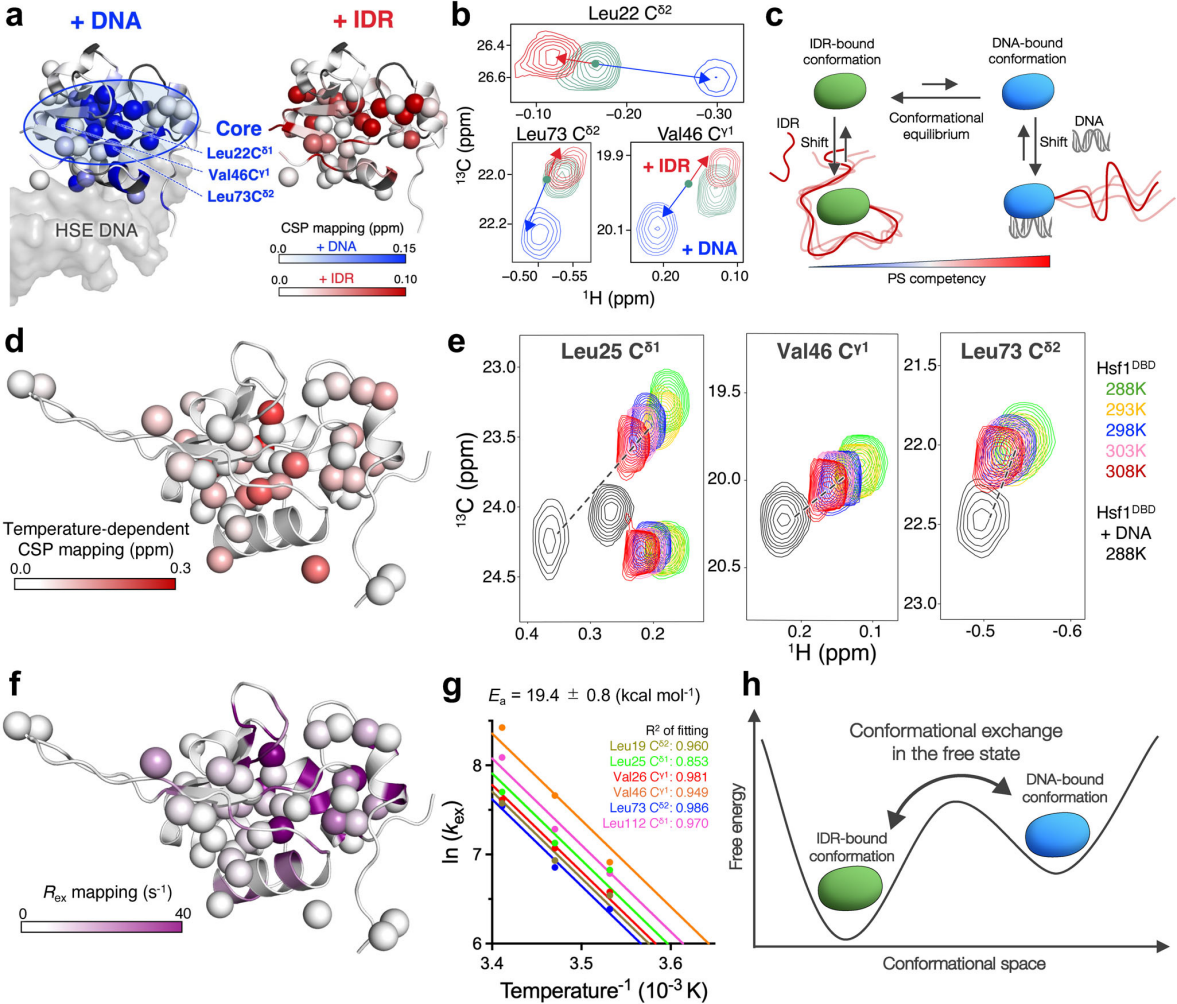
Conformational equilibrium of Hsf1^DBD^ coupled with DNA/IDR‐binding. (a) Mapping of the CSPs observed for the methyl resonances of Hsf1^DBD^ induced by the addition of DNA or Hsf1^IDR^. The structure of DBD is shown as the ribbon model with methyl groups shown as spheres (PDBID: 5D5V). HSE DNA is shown in gray surface model. (b) Representative resonances in ^1^H–^13^C HMQC spectra of Hsf1^DBD^ in the absence and presence of HSE DNA or Hsf1^IDR^, illustrating population shifts of conformational states induced by the ligand binding. DNA‐bound and IDR‐bound peaks are connected by a dashed line. (c) Schematic representation of conformational switching of Hsf1 coupled with DNA/IDR‐binding, as suggested by chemical shift analysis. DBD exists in equilibrium between DNA‐bound and IDR‐bound conformations. (d) Mapping of the temperature‐dependent CSPs observed for the methyl resonances of Hsf1^DBD^. The resonances at 288 and 308 K are compared. (e) Overlaid spectra of the Hsf1^DBD^ in the ligand‐free state at various temperatures and DNA‐bound DBD at 288 K. (f) Mapping of *R*_ex_ values obtained from ^15^N relaxation dispersion analysis on Hsf1^DBD^. The residues that showed substantial *R*_ex_ (>1 s^−1^) contributions to the transverse relaxation of the ^13^C methyl moiety and ^15^N from backbone amide moiety are mapped on the DBD structure. (g) *k*_ex_ of methyl moieties are plotted following the Arrhenius equation at different temperatures. Data are plotted only when the amplitudes of their relaxation dispersion profiles are well above the experimental errors. (h) Schematic representation of the energy landscape of the conformational equilibrium of Hsf1^DBD^ illustrated based on the information from dynamics analysis by NMR.

The effect of DNA binding to Hsf1 was investigated by NMR spectroscopy in diluted state. NMR analysis of the ^2^H^15^N‐labeled Hsf1^DBD–IDR^ showed that the signals from IDR were stronger than those from DBD, highlighting the highly mobile nature of IDR (Figure 2a). Importantly, the resonances from IDR in Hsf1^DBD–IDR^ showed reasonable correspondence with those from isolated Hsf1^IDR^, but several resonances showed significant differences, suggesting interdomain interactions (Figure 2a). Notably, size exclusion chromatography–multi angle light scattering (SEC–MALS) showed that both Hsf1^DBD–IDR^ and Hsf1^IDR^ existed as monodispersed monomers under NMR conditions, suggesting that these NMR signal changes primarily reflect intramolecular interactions (Figure S2). The regions recognized by DBD were defined based on chemical shift differences (CSDs) between Hsf1^DBD–IDR^ and Hsf1^IDR^ (Figure 2b). Analysis of the residue types in the DBD‐binding sites within the IDR revealed that aromatic and hydrophobic aliphatic residues are highly populated. On the other hand, negatively charged and polar residues are also highly populated, suggesting that the DBD–IDR interaction is supported by a complex combination of residues with diverse physicochemical properties (Figures 2c, S3a, and S4a–c). Given that PS of the Hsf1^IDR^ proceeds through hydrophobic driving forces [27], these findings suggest that DBD suppresses PS by sequestering key hydrophobic residues of IDR that are otherwise critical for mediating intermolecular interactions. Addition of DNA to Hsf1^DBD–IDR^ and Hsf1^DBD^ induced significant perturbations to the resonances from DBD, confirming the direct binding of DNA to DBD (Figures 3a and S5). Interestingly, DNA binding to Hsf1^DBD–IDR^ shifted the IDR resonances toward those of the isolated Hsf1^IDR^ (Figure 2a,b), indicating that interactions between IDR and DBD diminished upon DNA binding (Figure 2b). These phenomena were further validated by NMR measurement for ^2^H^15^N‐labeled Hsf1^IDR^: The perturbations from the resonances from Hsf1^IDR^ by the addition of Hsf1^DBD^ were diminished in the presence of HSE DNA (Figure S3b,d,e). Importantly, the lack of signal changes in Hsf1^IDR^ by the addition of HSE DNA confirmed that direct IDR–DNA interactions, if any, are much weaker or more transient than those between the DBD and DNA (Figure S3c,f). In addition, as the population of the DBD‐bound state increased from ^2^H^15^N‐labeled Hsf1^IDR^ to in the presence of Hsf1^DBD^ and then to ^2^H^15^N‐labeled Hsf1^DBD–IDR^, the NMR signals changed in a fast‐exchange manner (Figure S3g), suggesting that the DBD and IDR undergo binding and dissociation on the microsecond‐to‐millisecond timescale or faster [39]. Thus, these results suggest that DNA binding to DBD promotes the release of IDR from DBD.

Release of IDR from DNA‐bound DBD was supported by tryptophan–dansyl Förster resonance energy transfer (FRET) [40] and paramagnetic relaxation enhancement (PRE) experiments. In the FRET measurement, a dansyl‐maleimide was conjugated to the S485C mutation site in Hsf1^DBD‐IDR^, and energy transfer with tryptophan residues in DBD (Trp23, Trp37) was monitored (Figure 2d). The FRET efficiency decreased substantially from 0.18 to 0.07 upon addition of 1 equiv DNA, indicating the DNA binding induced dissociation of IDR from DBD (Figures 2e and S6). In PRE measurement, we designed the Hsf1^DBD‐IDR^ C36S/C373S/C378S construct retaining only one Cys residue in DBD (Cys103) for conjugation of (1‐oxyl‐2,2,5,5‐tetramethylpyrroline‐3‐methyl)‐methanethiosulfonate (MTSL) (Figure 2d). PRE causes signal intensity reduction depending on the distance between the MTSL's unpaired electron and observed nuclei [41]. The significant PRE‐induced intensity reductions were observed in several regions in IDR without DNA, however, these PREs were largely abolished upon DNA addition (Figures 2f and S7), strongly supporting the notion that DNA binding disrupts the interdomain interactions between DBD and IDR. Based on these results, we propose a scheme where DNA binding disrupts Hsf1^DBD–IDR^ interdomain interactions, leading to IDR release, which enables intermolecular IDR interactions promoting PS (Figure 2g).

### 2.3 Conformational Equilibrium of the DBD Tuned by the Interaction With DNA and IDR

To understand the mechanism of competition between DNA and IDR for DBD, we examined the mode of interaction between DBD and DNA or IDR by NMR titration experiments. Addition of DNA to the isolated Hsf1^DBD^ induced substantial chemical shift perturbations (CSPs) in intermediate exchange for residues within the α‐helices that directly contact HSE DNA (Figures 3a, S8a,b,d,f, and S9a). CSPs were also induced at the core regions of DBD that are distal from the DNA‐binding interface (Figures 3a, S8a,b, and S9a). These CSPs observed outside the direct DNA‐binding sites suggest that DNA binding affects the conformational state throughout DBD. Upon addition of Hsf1^IDR^ to the Hsf1^DBD^, CSPs were observed not only at the DNA‐binding interface but also across multiple other regions, suggesting that DNA‐induced release of IDR from the DBD is not driven solely by a simple direct competition mechanism (Figures 3a, S4d,e, S8a,c,e,f, and S9b). Notably, CSPs were also observed from the core upon the addition of Hsf1^IDR^, indicating that IDR binding also affects the conformational state in this core DBD region (Figure 3a, S8a,c,e,f, and S9b). Several signals displayed linear CSP trajectories between DNA‐bound, free, and IDR‐bound states (Figure 3b), a pattern remained consistent when comparing Hsf1^DBD–IDR^ plus DNA with isolated Hsf1^DBD^ (Figure S8g). This linearity indicates that even in its ligand‐free state, DBD exists in a two‐state conformational equilibrium between DNA‐ and IDR‐bound conformations as minor and major state, respectively (Figure 3c). These findings suggest a mechanism where DNA binding shifts conformational equilibrium of DBD, leading to allosteric IDR release from DBD.

Next, we tracked NMR signal changes of the Hsf1^DBD^ at varying temperature, aiming to perturb the equilibrium state [42]. Several methyl resonances from the isolated Hsf1^DBD^ showed temperature‐dependent CSPs (Figure S10a–c). Residues with temperature‐dependent CSPs were distributed throughout the DBD structure, including the core region that showed signal changes upon DNA or IDR binding (Figure 3a,d). As temperature increased, these methyl signals of the ligand‐free Hsf1^DBD^ progressively shifted toward the signals of the DNA‐bound state (Figure 3e). The observation of a linear van't Hoff's relationship across this temperature range strongly suggests a two‐state equilibrium [43], with higher temperatures favoring the DNA‐bound conformation (Figure S10d).

We further validated the kinetics of this conformational equilibrium through Carr–Purcell–Meiboom–Gill relaxation dispersion (CPMG RD) experiments [44] on Hsf1^DBD^. Both ^15^N‐backbone and ^13^C‐methyl CPMG RD measurements revealed substantial *R*_ex_ contributions (>1 s^−1^) to *R*_2_ across an extensive region of DBD, spanning from the DNA‐binding interface through the core region to the opposite face (Figures 3f and S11a). In the temperature range of 283–295 K, the exchange rate (*k*_ex_) values were in the order of ∼1000 s^−1^ (Figure S11b). Based on *k*_ex_ values at different temperatures, Arrhenius and Eyring analysis were performed to estimate the activation energy (*E*_a_) and the apparent activation free energy (Δ*G* ^‡^), respectively. These analyses yielded *E*_a_ = 19.4 kcal mol^−1^ and Δ*G* ^‡^ = 12.3 kcal mol^−1^ at 310 K (Figures 3g and S11c,d). This *E*_a_ coincides with the typical interaction energy of several hydrogen bonds or π–π stacking [45, 46, 47, 48], suggesting that the exchange involves concerted conformational rearrangement of DBD. Together, the temperature‐dependent CSPs and CPMG RD data provide compelling evidence for a two‐state conformational exchange between DBD‐bound and IDR‐bound conformations even in the ligand‐free state of DBD (Figure 3h). These findings suggest that elevated temperatures shift DBD toward its DNA‐bound conformation, becoming more prone to release IDR into an “PS‐competent state.” Although Hsf1 droplet formation is known to occur upon heat shock in the cell [28, 29], and recent reports show that PS of certain IDR regions is also temperature‐dependent [27], our findings suggest that the population shift in DBD's conformational equilibrium may also contribute to PS promotion under heat shock.

### 2.4 DNA Binding Modulates Fast Internal Dynamics of the DBD

**FIGURE 4:**
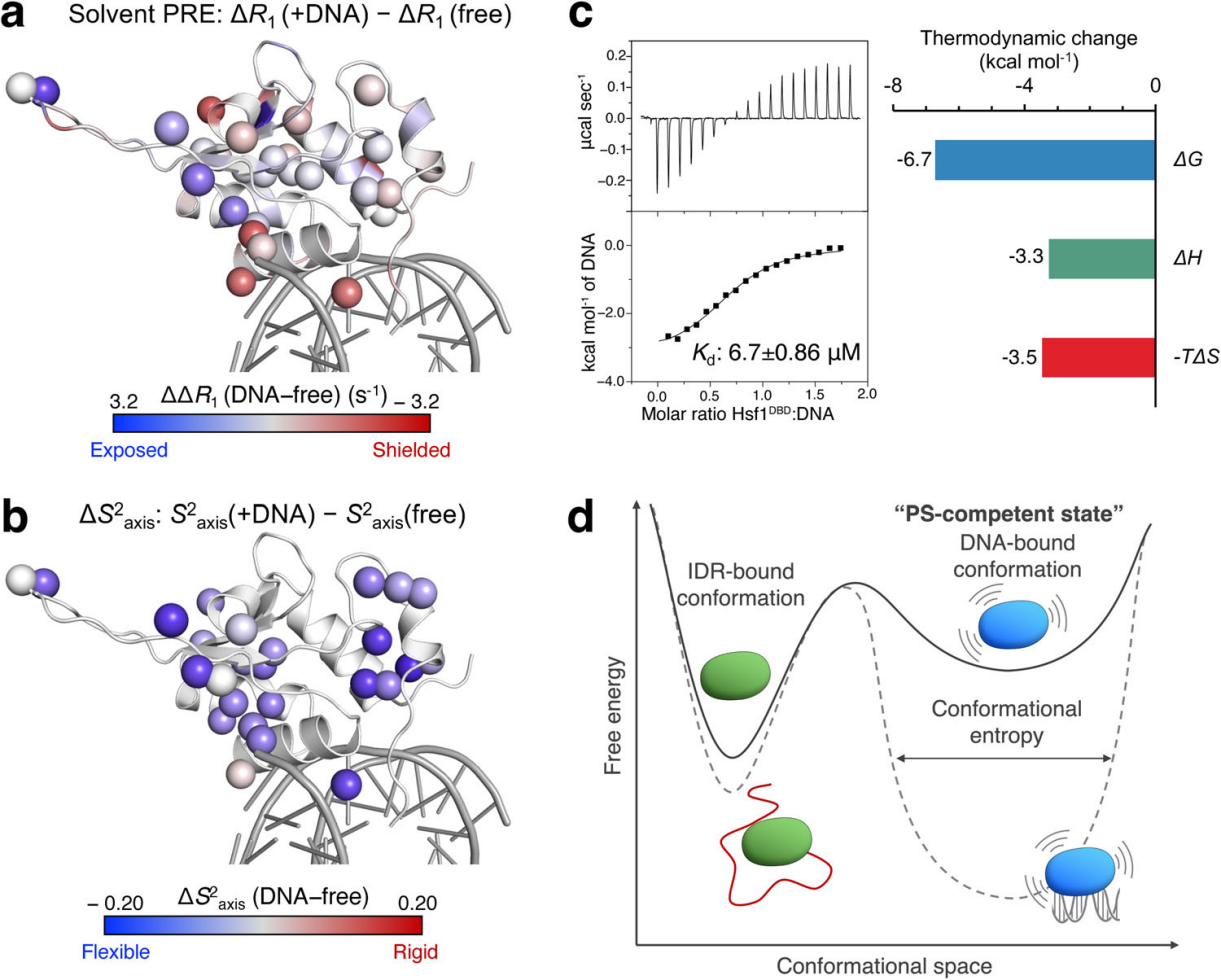
Fast internal dynamics of Hsf1^DBD^ modulated by DNA binding. (a) Mapping of the differential sPRE (ΔΔ*R*_1_) of Hsf1^DBD^ between the absence and presence of DNA. Positive values indicate increased solvent exposure upon DNA binding, whereas negative values indicate protection from solvent. (b) Mapping of the effect of DNA binding on the methyl order parameters, *S* ^2^_axis_, of DBD. The difference of methyl order parameters, Δ*S* ^2^_axis_, between DNA‐bound and apo states are mapped. In panels (a) and (b), the methyl groups used as probes in the analysis are shown as spheres. (c) ITC binding isotherms of the calorimetric titration of a specific DNA sequence to the DBD and the associated thermodynamic components (Δ*G*, Δ*H*, and −*T*Δ*S*) displayed as bars. (d) Schematic energy landscape of the DBD conformational equilibrium. The crystal structures of DBD in the free state and DNA‐bound states appear essentially identical, but the NMR analysis suggested that these two states differ in conformational entropy, meaning they represent states with different degrees of fluctuation.

Although previous crystal structures revealed that the overall three‐dimensional DBD structure remains largely unchanged upon DNA binding [17, 18], our NMR data on Hsf1^DBD^ suggested exchange between two distinct conformational states (Figure 3). Changes of the conformational state of the DBD upon DNA binding were investigated by solvent paramagnetic relaxation enhancement (sPRE) analysis—a sensitive probe of both transient and stable solvent exposure patterns [49] using the isolated Hsf1^DBD^. We evaluated sPRE based on the longitudinal relaxation rates (*R*_1_), using saturation recovery experiments conducted in the absence and presence of paramagnetic co‐solute DTPA‐BMA‐Gd (III) (Gd^3+^) [50, 51]. ^1^H–^13^C‐methyl and ^1^H–^15^N‐backbone signal intensities were recorded at multiple recovery delays and fitted to a single‐exponential functions to extract *R*_1_ values (Figures S12–S14). Paramagnetic contribution to *R*_1_, Δ*R*_1_, in the absence of DNA showed good correspondence with solvent‐accessible surface area (SASA) values calculated from crystal structures, validating sPRE as a reporter of solvent accessibility (Figure S12). We also measured sPRE for DNA‐bound Hsf1^DBD^ (Figure S13) and calculated the differences between the ligand‐free and DNA‐bound states, ΔΔ*R*_1_ values. The DNA‐binding interface had negative ΔΔ*R*_1_ values, indicating reduced solvent exposure (Figures 4a and S14). Remarkably, significant changes were detected also at sites distal from the DNA‐binding surface, including regions with reduced or increased solvent exposure (Figures 4a and S14). This result suggests that DNA binding triggers a population shift within the conformational ensemble, favoring states with altered dynamics of transient solvent exposure.

The effect of DNA binding on the conformational dynamics of the DBD was further evaluated by methyl group *S* ^2^ order parameters (*S* ^2^_axis_) as quantified by ^1^H spin‐based relaxation‐violated triple‐quantum coherence transfer NMR spectroscopy [52, 53]. DNA binding to the DBD notably decreased *S* ^2^_axis_, indicating increased amplitude of ps–ns dynamics throughout the protein (Figures 4b and S15). Although the methyl groups in the vicinity of the DNA‐binding interface also showed negative Δ*S* ^2^_axis_ values, this observation is consistent with the interaction modes seen in the crystal structure of Hsf1^DBD^‐DNA complex [17]. In this complex, the DNA is recognized primarily by charged residues, such as Arg71 and Arg79, whereas the methyl groups have no major contact with DNA (Figure S15f). This observation is highly consistent with the data from isothermal titration calorimetry (ITC), showing that DBD‐DNA association is driven by both enthalpic and entropic contributions (Figure 4c). Collectively, the data support a model in which the DBD samples an equilibrium between conformational states with distinct fluctuation patterns, and DNA binding allosterically biases this equilibrium toward the more dynamic state (Figure 4d).

### 2.5 Allosteric Mutation on DBD Induces an Equilibrium Shift to PS‐Competent State

**FIGURE 5:**
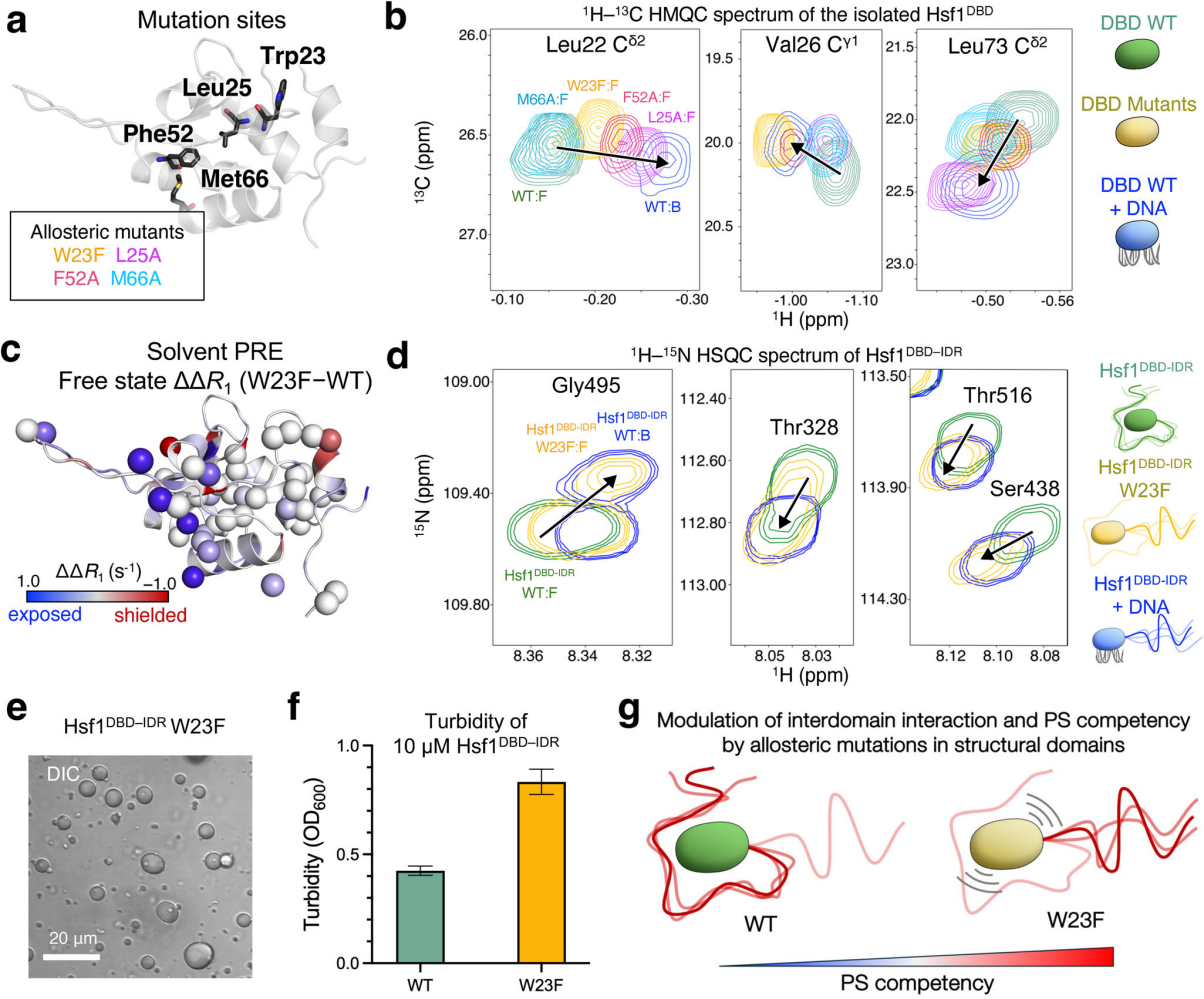
Modulation of DBD conformational equilibrium alters PS competency. (a) Structure of Hsf1^DBD^ showing the amino acid residues mutated to Ala in stick representation. (b) Representative signals on ^1^H–^13^C HMQC spectra of the isolated Hsf1^DBD^ WT (WT:F), W23F (W23F:F), L25A (L25A:F), F52A (F52A:F), M66A (M66A:F), and DNA‐bound Hsf1^DBD^ WT (WT:B). The resonances of the mutants in the apo state are located in between those of the wild‐type in apo and DNA‐bound states, indicating that the mutations shift the conformational equilibrium toward the DNA‐bound state. (c) Mapping of the differential sPRE between Hsf1^DBD^ and Hsf1^DBD^ W23F as represented by ΔΔ*R*_1_. The Methyl groups that were used as probes in the analysis are shown as spheres. (d) Representative signals on ^1^H–^15^N HSQC spectra of free Hsf1^DBD‐IDR^ (WT:F), Hsf1^DBD–IDR^ in complex with HSE DNA (WT:B), and free Hsf1^DBD–IDR^ W23F (W23F:F). For clarity, only the first three counters are shown. (e) DIC images of Hsf1^DBD–IDR^ W23F droplets. The images were acquired at 20 min after induction of PS by reduced pH to 4.5. (f) Evaluation of the W23F mutation effect on PS using the turbidity assay. The significantly higher turbidity observed for the W23F mutant indicates enhanced formation of phase‐separated condensates. The turbidity was measured at 5 min after induction of PS by reduced pH to 4.5. Error bars represent standard deviation from three independent measurements. (g) Schematic representation of the effect of the W23F mutation to conformational states of Hsf1. The W23F mutation allosterically modulate the dynamics of DBD, resulting release of IDR.

Our multiscale analysis suggested an interconnected regulatory mechanism linking side chain dynamics to global Hsf1^DBD–IDR^ conformational transitions. This mechanistic insight guided us to design mutants targeting the allosteric network within DBD. We focused on several residues forming the core of DBD and generated Phe or Ala substitution mutants to systematically alter the steric bulk at this position (Figure 5a,b). ^1^H–^13^C HMQC spectra of the Hsf1^DBD^ mutants revealed that signals from mutants in the free state were positioned in the middle between signals from wild‐type free state and DNA‐bound state (Figure 5b), indicating equilibrium shift toward the DNA‐bound conformation. Further analysis of the Hsf1^DBD^ W23F mutant revealed that the mutation induced extensive CSPs throughout the protein, demonstrating that this single‐point mutation triggered domain‐wide conformational redistribution (Figure S16a–e). The overall fold appeared to be maintained, as signals of Hsf1^DBD^ W23F were on the line connecting free and DNA‐bound signals of wild‐type DBD, and CD measurements further showed that the melting temperature was largely unchanged (Figure S16f). sPRE measurements of the isolated Hsf1^DBD^ W23F in the ligand‐free state revealed increased ΔΔ*R*_1_ values, indicating enhanced solvent exposure consistent with increased population of the DNA‐bound form observed in the CSP analysis (Figures 5c and S17).

Given this increased population of the DNA‐bound conformation conferred by the W23F mutation, we evaluated whether this mutation facilitated IDR release from the DBD. Following the approach described in Figure 2a, we compared NMR signals from IDR in Hsf1^DBD‐IDR^ before and after DNA addition with those from Hsf1^DBD‐IDR^ W23F. The mutants showed the signals from IDR shifted toward those of DNA‐bound Hsf1^DBD‐IDR^ (Figures 5d and S18), suggesting that W23F mutations promoted IDR release even without DNA. We then performed a PS assay, which showed that Hsf1^DBD–IDR^ W23F formed spherical droplets (Figure 5e), and exhibited higher turbidity at 10 µM compared with the wild‐type construct (Figure 5f). These findings indicate that allosteric mutation in structured DBD propagates through the conformational equilibrium to drive global Hsf1^DBD‐IDR^ rearrangements and PS competency, definitively linking local dynamics to PS in Hsf1 (Figure 5g).

## 3 Conclusion

In this study, we elucidated the molecular mechanism regulating PS through the conformational dynamics of DBD. Our data show that DNA binding to DBD drives the transition from an autoinhibited state, in which DBD–IDR interactions suppress assembly, to a PS‐competent state, in which IDR is released. Previously, stress‐dependent transcriptional activation of Hsf1 was thought to be governed mainly by monomer–oligomer transitions and condensate formation through PS [11, 24, 29]. The monomer–oligomer transition is regulated by switching between intramolecular interactions of LZ4 in IDR with LZ1–3 and intermolecular interactions of LZ1–3, whereas PS is primarily driven by intermolecular IDR–IDR interactions [20, 21, 23, 27]. Our findings reveal that intramolecular DBD–IDR interactions function as an autoinhibitory module of PS. DBD appears to primarily capture hydrophobic residues within IDR, which are critical for lower critical solution temperature type PS of Hsf1 [27], thereby suppressing nonspecific interactions (Figure 2). Our data suggest that these intramolecular DBD–IDR interactions act as a molecular switch that keeps Hsf1 in an autoinhibited state until it interacts with HSE (Figure 2). Many transcription factors share common domain architecture consisting of a structured DBD and a flexible IDR [3, 4]. Intramolecular DBD–IDR interactions are increasingly recognized as regulators of higher‐order functions such as DNA‐binding specificity and affinity [6, 32, 33, 34, 35, 36, 37, 38]. Our study extends the functional relevance of this architecture in the PS regulation, underpinning robust responses to stress with minimal non‐specific PS outside the specific binding motif.

Conventionally, DNA‐mediated PS enhancement has been attributed to the accumulation of proteins using DNA as a molecular scaffold [54, 55]. Our findings reveal a fundamentally different mechanism in which DNA functions as a trigger by controlling conformational dynamics across multiple scales, from motions of DBD side chains to domain‐level rearrangements (Figures 2, 3, 4). This DNA‐responsive conformational switching provides a molecular basis for context‐dependent PS regulation, where PS occurs specifically when Hsf1 encounters its cognate DNA sequences rather than through non‐specific protein accumulation. Furthermore, although the heat response of Hsf1 has generally been attributed to its IDR, we found that the structured DBD itself—traditionally regarded as a static molecular receptor owing to its relatively rigid architecture [56, 57]—also functions as a thermosensor through conformational shifts (Figures 3, 4, 5). This observation not only reinforces the sophisticated regulatory mechanism of Hsf1 as a heat‐responsive transcription factor, but also challenges the conventional view of DBDs as passive DNA‐binding modules and reveals their active role in regulating PS properties.

Although fluctuations across multiple timescales have been reported for several other DNA‐binding proteins [58, 59, 60, 61], our study provides crucial mechanistic insights by directly bridging such conformational dynamics to the functional output of PS. We further revealed that allosteric networks within DBD govern its dynamic conformational equilibrium, which in turn regulates intermolecular interactions and PS properties. These findings establish a dynamics‐function relationship where DBD conformational dynamics directly determines the accessibility of IDRs for PS, providing a molecular explanation for the specificity of transcription factor PS. From a therapeutic perspective, although drug discovery targeting IDRs has garnered significant interest [62, 63], technical challenges remain formidable because IDRs lack well‐defined structures. Our identification of allosteric networks within DBD suggests alternative therapeutic strategies that could modulate PS through targeting structured domains. Unlike direct inhibition of DNA binding, which carries inherent risks of off‐target effects [64, 65, 66], allosteric modulation of DBD dynamics could potentially fine‐tune transcription factor activity without completely disrupting essential DNA‐protein interactions. This approach may offer new opportunities for therapeutic intervention in diseases characterized by dysregulated stress responses or aberrant PS.

## Author Contributions

**Soichiro Kawagoe**: conceptualization, investigation, funding acquisition, writing – original draft, visualization, formal analysis, data curation, project administration, resources, writing – review and editing, methodology. **Hiroyuki Kumeta**: writing – review and editing, investigation, data curation, methodology, formal analysis, resources. **Tomohide Saio**: conceptualization, funding acquisition, writing – original draft, writing – review and editing, project administration, supervision, data curation, visualization, resources.

## Funding

JSPS KAKENHI (JP23K19353 (SK), JP24K18063 (SK), JP23H05470 (TS), JP23K23824 (TS), JP23K26688 (TS), JP25K02217 (TS)), JST FOREST (JPMJFR204W (TS)), JST ASPIRE (JPMJAP2526 (TS)), MEXT Grant‐in‐Aid for Transformative Research Areas (B) JP21H05094 and JP21H05093 (TS), the Astellas Foundation for Research on Metabolic Disorders (SK, TS), the Takeda Science Foundation Grant (TS), Asahi Glass Foundation (TS), the Mochida Memorial Foundation for Medical and Pharmaceutical Research (TS), Naito Foundation, (TS), Serika Fund (TS), the Nakajima Foundation (TS), the Canon Foundation (TS), JKA Foundation (TS), and JSPS Program for Forming Japan's Peak Research Universities (J‐PEAKS) (JPJS00420240022 (SK, TS)).

## Conflicts of Interest

The authors declare no conflicts of interest.

## Supporting information



Fluorescence and DIC image of droplets, SEC‐MALS profile, NMR spectra, FRET, CPMG RD profile, ITC, sPRE, fast motion dynamics, and CD spectroscopy.
**Supporting File**: anie72666‐sup‐0001‐SuppMat.pdf.

## Data Availability

The data that support the findings of this study are available from the corresponding author upon reasonable request.
