# Supplementary material for: DNA‐Induced Entropic Gain Triggers an Allosteric Switch for Biomolecular Condensation of Heat Shock Transcription Factor 1
Source: Angew Chem Int Ed Engl. 2026 May 19;65(28):e7340537. doi: 10.1002/anie.7340537 (PMC13340490; doi:10.1002/anie.7340537)
Supplement: Supplementary file 1 — Fluorescence and DIC image of droplets, SEC‐MALS profile, NMR spectra, FRET, CPMG RD profile, ITC, sPRE, fast motion dynamics, and CD spectroscopy. Supporting File: anie72666‐sup‐0001‐SuppMat.pdf. [file ANIE-65-e7340537-s001.pdf]

Supporting Information

DNA-Induced Entropic Gain Triggers an Allosteric Switch for Biomolecular Condensation of Heat Shock Transcription Factor 1

Soichiro Kawagoe*[a] [b], Hiroyuki Kumeta[c], Tomohide Saio*[a] [b] [d]

[a] Dr. S. Kawagoe, Prof. T. Saio
Institute of Advanced Medical Sciences
Tokushima University
Tokushima 770-8503, Japan

[b] Dr. S. Kawagoe, Prof. T. Saio
Institute of Photonics and Human Health Frontier
Tokushima University
Tokushima 770-8501, Japan

[c] Dr. H. Kumeta
Faculty of Advanced Life Science
Hokkaido University
Hokkaido 060-0810, Japan

[d] Prof. T. Saio
Fujii Memorial Institute of Medical Sciences
Tokushima University
Tokushima, 770-8503, Japan

*Email: kawagoe@tokushima-u.ac.jp, saio@tokushima-u.ac.jp

Materials and Methods

Expression and purification of protein samples

Human Hsf1 (1–529), Hsf1^{DBD} (1–120), Hsf1^{IDR} (209–529), Hsf1$^{DBD\text{-}IDR}$ (Δ121–208), Hsf1$^{DBD\text{-}IDR}$ C36S/C373S/C378S, Hsf1$^{DBD\text{-}IDR}$ S485C/C36S/C103S/C373S/C378S, Hsf1-mCherry, Hsf1$^{DBD\text{-}IDR}$-mCherry, and Hsf1^{DBD} mutants (W23F, L24A, F52A, M66A), expression constructs were cloned into the pET21b vector (Cat. No. 69741-3CN; Novagen, Madison, WI, USA) and fused to GB1-His^6 tags at the HRV3C N-terminus of the protease cleavage site. All mutants were constructed through site-directed mutagenesis using the PrimeSTAR Mutagenesis Basal Kit (Cat. No. R046A; Takara Bio, Shiga, Japan). All expression constructs were transformed into BL21 (DE3) cells. The cells were grown in the Luria–Bertani medium at 37 °C in the presence of 50 μg mL^{-1} ampicillin. Subsequently, protein expression was induced by adding 0.5 mM isopropyl-β-D-1-thiogalactopyranoside (IPTG) at OD_{600} ~0.6, followed by a 12–16 h incubation at 18 °C. Then, the cells were harvested at OD_{600} ~3.0, resuspended in a lysis buffer containing 50 mM Tris-HCl (pH 8.0) and 500 mM NaCl, disrupted by a sonicator, and centrifuged at 18,000 rpm for 30 min. The supernatant fraction containing Hsf1 was purified using an Ni-NTA Sepharose column (Cat. No. 30210; Qiagen, Hilden, Germany). Additionally, the GB1-His^6 tag was removed using a HRV3C protease at 4 °C (incubation for 16 h), and the cleaved Hsf1 was applied onto a HiTrap Q HP anion exchange column (Cat. No. 17115401; Cytiva, Tokyo, Japan), pre-equilibrated with 25 mM HEPES/NaOH (pH 7.5), 10% glycerol, 20 mM NaCl, and eluted with a linear gradient of 20–500 mM NaCl. Samples were further purified by gel filtration using a Superdex 200 pg 16/600 column (Cat. No. 28989335; Cytiva) or Superdex 75 pg 16/600 column (Cat. No. 28989333; Cytiva) equilibrated with a solution containing 25 mM MES/NaOH (pH 5.5), 100 mM NaCl, and 0.02% NaN_3. Finally, protein concentrations were determined spectrophotometrically at 280 nm using the corresponding extinction coefficient.

Protein isotope labeling for NMR studies

For isotope labeling of Hsf1^{IDR} and Hsf1$^{DBD\text{-}IDR}$, M9 medium containing $^{15}NH_4Cl$ and [^{2}H,^{12}C]-glucose in 99.9% D_2O was used. Hsf1^{DBD} was expressed using the same deuterated medium or M9 medium containing $^{15}NH_4Cl$ and [^{1}H,^{12}C]-glucose in H_2O. To obtain Ile-δ^1-[$^{13}CH_3$], Val,Leu-[$^{13}CH_3$,$^{12}CD_3$], Met-[$^{13}CH_3$], and Ala-[$^{13}CH_3$] Hsf1^{DBD}, alpha-ketobutyric acid [methyl-$^{13}CH_3$] (50 mg liter^{-1}), alpha-ketoisovaleric acid [monothyl-$^{13}CH_3$,$^{12}CD_3$] (85 mg liter^{-1}), [$^{13}CH_3$]-Met (50 mg liter^{-1}), and [D_2, $^{13}CH_3$]-Ala (50 mg liter^{-1}) were added to the culture 0.5 h prior to the addition of IPTG. Protein expression was induced by adding 0.5 mM IPTG at OD_{600} ~0.6, followed by 12−16 h of incubation at 18 °C. The cells were harvested at OD_{600} ~2.0, resuspended in lysis buffer containing 50 mM Tris-HCl (pH 8.0) and 500 mM NaCl, and stored at −80 °C. These samples were purified using the same procedure as when cultured in Luria–Bertani medium.

Turbidity assay

For evaluating the effect of HSE DNA on PS of Hsf1$^{DBD\text{-}IDR}$, varying molar equivalents of DNA were added, and the mixture was diluted with a solution containing 25 mM CH3COONa (pH 4.5), 100 mM NaCl, and 0.02% NaN_3 to achieve a final concentration of 5 μM Hsf1$^{DBD\text{-}IDR}$. After incubation at 20 °C for 5 min, OD_{600} was measured using a JASCO V-730BIO spectrophotometer. Measurements were performed in triplicate, and standard deviations were used as error bars. For Hsf1 oligomer, turbidity measurements were additionally carried out in 25 mM HEPES-KOH (pH 7.2), 100 mM KCl, 2 mM $MgCl_2$, and 10 mM DTT, in the absence or presence of 0 or 4 repeat

HSE DNA. For evaluating the W23F allosteric mutation, the samples were diluted to achieve arbitrary final protein concentrations, incubated, and measured following the same procedure.

Confocal microscopy

To prepare droplets, 36 μM Hsf1 and 4 μM Hsf1-mCherry or 36 μM Hsf1$^{DBD\text{-}IDR}$ and 4 μM Hsf1$^{DBD\text{-}IDR}$-mCherry were mixed with 36 μM HSE DNA and 4 μM HSE DNA-6FAM. The double-stranded DNA was prepared by annealing 5′-gcgcgcgaGAAtgcgcgcg-3′ and 5′-cgcgcgcaTTCtcgcgcgc-3′. DIC and fluorescence images of droplets were obtained using a confocal microscope (FV1200, Olympus, Tokyo, Japan) equipped with a UPLSAPO 40X2 objective lens (NA 0.95). The buffer contained 25 mM CH_3COONa (pH 4.5), 100 mM NaCl, and 0.02% NaN_3. For DIC imaging under crowding conditions, 40 μM samples were used. Hsf1 oligomer droplets were formed in 25 mM HEPES-KOH (pH 7.2), 100 mM KCl, 2 mM $MgCl_2$ and 10 mM DTT in the presence of 5% (w/v) ficoll 400 or 5% (w/v) dextran 200, or in 25 mM MES-NaOH (pH 5.5), 100 mM NaCl, and 0.02% NaN_3. Hsf1$^{DBD\text{-}IDR}$ WT and W23F droplets were formed in 25 mM CH_3COONa (pH 4.5), 100 mM NaCl, and 0.02% NaN_3, and DIC images were acquired under the same microscope settings.

FRET measurement

Fluorescence measurements were performed using a JASCO FP-8300 spectrofluorometer and using solutions prepared in 25 mM MES/NaOH pH 5.5, 100 mM NaCl, and 0.02% NaN_3. The concentration of the dansyl-maleimide-modified Hsf1$^{DBD\text{-}IDR}$ S485C/C36S/C103S/C373S/C378S solution was 10 μM. The measurements were conducted at 298 K. The unpolarized emission spectra of tryptophan were recorded between 300 and 700 nm using an excitation wavelength of 295 nm and scan speed of 200 nm/min. The excitation and emission slit widths were both set to 5 nm. Each spectrum represented an integration of three consecutive scans. The energy transfer efficiency, E, was calculated for each DNA concentration using the fluorescence intensity at 320 nm of dansyl-modified Hsf1$^{DBD\text{-}IDR}$ S485C (F_{DA}) and without modification (F_{D}) as follows:

$$E = 1 - \frac{F_{\mathrm{DA}}}{F_{\mathrm{D}}} \quad (1)$$

SEC–MALS experiments

SEC–MALS was performed using DAWN HELEOS8+ (Wyatt Technology Corporation, Santa Barbara, CA, USA), a high-performance liquid chromatography pump (LC-20AD; Shimadzu, Kyoto, Japan), refractive index detector RID-20A (Shimadzu), and UV-vis detector SPD-20A (Shimadzu), which were located downstream of the Shimadzu liquid chromatography system connected to a PROTEIN KW-803 gel filtration column (Cat. No. F6989103; Shodex, Tokyo, Japan). Differential RI (Shimadzu) downstream of MALS was used to determine the protein concentrations. The running buffer used contained 25 mM MES-NaOH (pH 5.5), 100 mM NaCl, and 0.02% NaN_3. Approximately, 100 μL of a 50 μM sample was injected at a flow rate of 1.0 mL min^{-1}. Data were then analyzed using ASTRA version 7.0.1 (Wyatt Technology Corporation). Molar mass analysis was also performed over half of the width of the UV peak top height.

ITC experiment

Calorimetric titrations were carried out on a MicroCal VP-ITC microcalorimeter (Malvern Panalytical, Worcestershire, UK) at 283 K. All protein samples were purified in the ITC buffer containing 25 mM NaPi pH 7.2, 25 mM NaCl, and 0.02% NaN_3 by gel filtration. A 1,800 μL

sample cell was filled with a 60 μM Hsf1^{DBD} solution, and the 360 μL injection syringe was filled with a 0.5 mM solution of the HSE DNA containing one HSE motif. The titrations were carried out with a preliminary 2 μL injection, followed by 16 injections of 18 μL, each with a time interval of 3 min. The solution was stirred at 307 rpm. Data for the preliminary injection, which are affected by diffusion of the solution from and into the injection syringe during the initial equilibration period, were discarded. Binding isotherms were generated by plotting enthalpy values of reaction normalized by the modes of injectant versus the ratio of total injectant to total protein per injection. The data were fitted with Origin 7.0 (OriginLab Corporation).

NMR spectroscopy

NMR samples were prepared in 25 mM MES/NaOH (pH 5.5) and 100 mM NaCl, 0.02% NaN_3, and 10% D_2O. Concentration of Hsf1^{DBD} was adjusted to 400 μM. The NMR experiments were performed at 298 K. NMR spectra were obtained with a Bruker AVANCE NEO 800 MHz spectrometer (Bruker, Billerica, MA) using a CPTCI $^{1}H/^{19}F$-$^{13}C/^{15}N$ proton-optimized triple resonance cryoprobe and a Bruker AVANCE III 500 MHz spectrometer equipped with a BBFO probe. Backbone and methyl resonance assignments of the wild-type DBD were obtained from BMRB entry 17683. ^{15}N resonance assignments in IDR were carried out using the following set of the spectra measured at 298 K; ^{1}H–^{15}N HSQC, HNCO, HNCA, HN(CO)CA, HNCACB, CBCA(CO)NH. The ^{1}H, ^{15}N chemical shifts were referred to DSS according to the IUPAC recommendation. The data were processed using NMRPipe [1] and data analysis was performed with Olivia (https://github.com/yokochi47/Olivia). CSPs for backbone amide groups were calculated using the following equation:

$$\Delta\delta = \sqrt{\Delta\delta_{\mathrm{H}}{}^{2} + (0.2\Delta\delta_{\mathrm{N}})^{2}} \qquad (2)$$

where $\Delta\delta_{\mathrm{H}}$ and $\Delta\delta_{\mathrm{N}}$ are chemical shift changes of ^{1}H and ^{15}N, respectively. We defined regions in the IDR as DBD-binding sites when at least three out of five consecutive residues showed CSP values exceeding the average CSP threshold of 0.0126 ppm. CSPs for methyl groups were calculated using the following equation:

$$\Delta\delta = \sqrt{(\frac{\Delta\delta_{\mathrm{H}}}{\alpha})^{2} + (\frac{\Delta\delta_{\mathrm{C}}}{\beta})^{2}} \qquad (3)$$

where $\Delta\delta_{\mathrm{H}}$ and $\Delta\delta_{\mathrm{C}}$ are chemical shift changes of ^{1}H and ^{13}C, respectively, due to the addition of the ligand, and α and β are chemical shift distributions of ^{1}H and ^{13}C, respectively, of methyl groups as reported in the Biological Resonance Data Bank (http://www.bmrb.wisc.edu).

Paramagnetic relaxation enhancement experiment

To observe PRE, nitroxide spin label MTSL (Cat. No. O875000; Toronto Research Chemicals Inc., Toronto, ON, Canada) was introduced via cysteine-specific modification of Cys103 in Hsf1$^{DBD\text{-}IDR}$ C36S/C373S/C378S. Mutations in the C36S/C373S/C378S mutant and its MTSL derivatives were determined not to perturb the Hsf1$^{DBD\text{-}IDR}$ structure, as assessed by ^{1}H-^{15}N HSQC spectra. During purification, 10 mM DTT was added to [U-^{2}H;^{15}N]-labeled Hsf1$^{DBD\text{-}IDR}$ C36S/C373S/C378S, followed by incubation at room temperature for 10 min. The sample was then subjected to gel filtration using Superdex 200 pg 16/600 column, and the buffer was exchanged to a solution containing 25 mM HEPES-KOH (pH 7.2) and 100 mM KCl. MTSL was added from a concentrated stock in acetonitrile at a 10-fold excess, and the reaction was allowed to proceed at room temperature for ~6 h. Excess MTSL was extensively removed by an Amicon

stirred cell and exchanged into a solution containing 25 mM MES-NaOH (pH 5.5), 100 mM NaCl, and 0.02% NaN_3. PREs were observed from ^{1}H–^{15}N HSQC spectra of Hsf1$^{DBD\text{-}IDR}$ by measuring peak intensities before (paramagnetic) and after (diamagnetic) reduction of the nitroxide spin label by 5 mM ascorbic acid.

Solvent paramagnetic relaxation enhancement experiment

NMR spectra were recorded at recovery delays of 0.25, 0.50, 1, 2, and 3 s in the presence and absence of 2.0 mM DTPA-BMA-Gd (III) (Omniscan). Peak intensities were normalized such that the intensity at 0 s was set to 0, and the intensity at 3 s was set to 1. R_1 values were determined by curve fitting using equation (4). Fitting errors were used as error bars in the bar graph. The NMR experiments were performed at 298 K.

$$I = I_0\left(1 - exp^{(-R_1 t)}\right), \quad (4)$$

where I is the observed signal intensity at time t, I_0 is the plateau value (set to 1.0), R_1 is the longitudinal relaxation rate, t is the recovery time (recovery delay + acquisition time + fixed delay). These measurements were conducted both before and after the addition of 2 equivalents of DNA. The R_1 enhancement upon gadodiamide addition (ΔR_1) was calculated for each condition, and the difference between these values ($\Delta\Delta R_1$) was determined. Only $\Delta\Delta R_1$ values that exceeded the experimental error were considered significant and included in the analysis. The curve was analyzed using Prism 5 (GraphPad Software, San Diego, CA).

CPMG RD experiments

For the measurements of ^{15}N TROSY-based CPMG relaxation dispersion (RD) [2] and ^{13}C SQ CPMG RD [3,4], wild-type Hsf1^{DBD} were prepared at a 0.5 mM concentration in a solution containing 25 mM MES-NaOH (pH 5.5), 100 mM NaCl, and 0.02% NaN_3. NMR spectra were measured on a Bruker AVANCE Neo 800 MHz spectrometer equipped with a cryogenic TCI probe at 283, 288, and 298 K. CPMG pulses were applied at frequencies (νCPMG) of 25, 50, 100, 200, 300, 400, 600, 800, and 1,000 Hz during a 40 ms relaxation delay.

Two-site exchange rate constants (k_{ex}) were extracted using GUARDD [5] by fitting the data to the Carver–Richards–Jones all-timescales dispersion equation [6]. The activation energy (E_a) for conformational exchange was determined by fitting the temperature dependence of k_{ex} values to the Arrhenius equation (5):

$$\ln(k_{ex}) = \ln(A) - \frac{E_a}{RT} \quad (5)$$

where R is the universal gas constant (1.987 cal mol^{-1} K^{-1}) and T is the temperature in Kelvin. The slope was analyzed using Prism 5.

The apparent activation parameters for conformational exchange was determined by fitting the temperature dependence of k_{ex} values to the Eyring equation [7] (6):

$$\ln\left(\frac{k_{ex}}{T}\right) = -\frac{\Delta H^{\ddagger}}{R}\cdot\frac{1}{T} + \ln\left(\frac{k_B}{h}\right) + \frac{\Delta S^{\ddagger}}{R} \quad (6)$$

Where k_B is the Boltzmann constant, h is the Plank constant, $\Delta H^{\ddagger}$ is the apparent activation enthalpy, and $\Delta S^{\ddagger}$ is the apparent activation entropy. The apparent activation free energy at 310 K was then calculated using:

$$\Delta G^{\ddagger} = \Delta H^{\ddagger} - T\Delta S^{\ddagger} \quad (7)$$

Linear fitting was performed in GraphPad Prism by global fitting of the data from multiple residues. The mean $\Delta G^{\ddagger}$ and $\Delta S^{\ddagger}$ value derived from the individual residue-specific estimates is shown in Figure S11.

Fast internal motion (picosecond to nanosecond) dynamics of sidechain

To measure the order parameters of the methyl groups (S^2_{axis}) in Hsf1^{DBD}, we employed the ^{1}H spin-based relaxation violated coherence transfer NMR spectroscopy [8,9]. For this purpose, we used Hsf1^{DBD} in which the methyl groups of Ala, Ile, Leu, Met, and Val residues were $^{13}CH_3$-labeled. The intra-methyl ^{1}H–^{1}H dipolar cross-correlated relaxation rates η were obtained by fitting the ratios of the peak intensities measured in pairs of data sets (I_a and I_b) recorded as a function of relaxation time, T to the following equation:

$$\left|\frac{I_a}{I_b}\right| = C\frac{\eta \tan\mathrm{h}\left(\sqrt{\eta^2+\delta^2}T\right)}{\sqrt{\eta^2+\delta^2} - \delta \tan\mathrm{h}\left(\sqrt{\eta^2+\delta^2}T\right)} \quad (8)$$

where C=0.75 in this case, T is the varied delay, δ is a parameter related to the density of the external protons around the methyl group, whereas I_a and I_b are the intensities of the forbidden and allowed coherences, respectively, with delay T. The data sets were recorded using the following sets of relaxation delays T: 4, 8, 12, 16, 20, 24, 28, 32, 37, 41, 44, and 48 ms at 298 K. The recovery delay was set to 1.5 s.

S^2_{axis} values were calculated by the determined η value using the equation below:

$$\eta = \frac{R^F_{2,H} - R^S_{2,H}}{2} \approx \frac{9}{10}\left(\frac{\mu_0}{4\pi}\right)^2\left[P_2\left(\cos\theta_{axis,HH}\right)\right]^2\frac{S^2_{axis}\gamma_H^4\hbar^2\tau_c}{r^6_{HH}} \quad (9)$$

where τ_c is the rotational correlation time of Hsf1^{DBD}, $R^F_{2,H}$ and $R^S_{2,H}$ are the relaxation rates of fast and slowly relaxing coherences, respectively, γ_H is proton gyromagnetic ratio and r_{HH} is the distance between pairs of methyl protons. The rotational correlation times of Hsf1^{DBD} in the absence and presence of HSE DNA were predicted from the crystal structure using HydroPro [10].

CD spectroscopy

Thermal denaturation of Hsf1^{DBD} WT and W23F mutants was monitored by CD spectroscopy. Measurements were performed at a final concentration of 10 μM in 10 mM MES–NaOH (pH 5.5) containing 100 mM NaF, and the ellipticity at 222 nm was monitored using a bandwidth of 2 nm. Samples were heated from 25 to 80 °C at a rate of 0.5 °C min^{-1}, and data points were collected every 0.2 °C. The resulting thermal denaturation curves were fitted with a Boltzmann sigmoidal equation to determine the melting temperature [11].

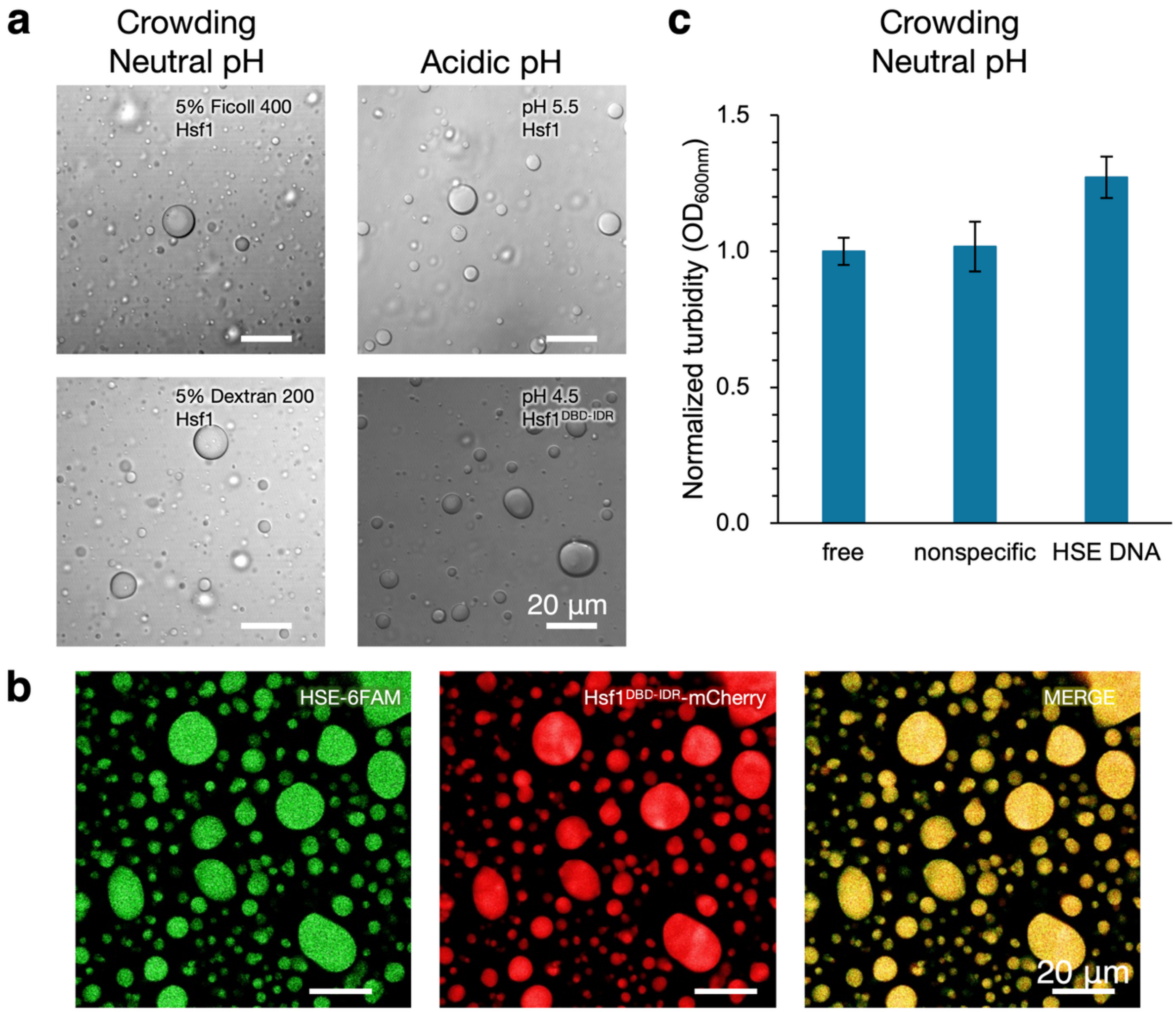

Figure S1. HSE DNA dissolves into the droplet of Hsf1. (a) Representative differential interference contrast image showing the droplets formed by Hsf1 in the presence of 5 (% w/v) ficoll 400 (upper left) or dextran 200 (lower left), and by Hsf1 or Hsf1$^{DBD\text{-}IDR}$ under acidic condition at pH 5.5 (upper right) or pH 4.5 (lower right), respectively. (b) Fluorescence microscope images of Hsf1$^{DBD\text{-}IDR}$ (with 0.1 eq Hsf1$^{DBD–IDR}$–mCherry) and 1 repeat HSE DNA (with 0.1 eq 6-FAM-DNA). (c) Turbidity assay for Hsf1 oligomer in the absence (free) and presence of DNA containing 0 (nonspecific) and 4 repeat HSE at pH 7.2, showing the enhanced droplet formation of Hsf1 induced by HSE DNA. Error bars represent the standard deviation from three independent measurements. The turbidity was measured at 5 min after induction of PS by adding 10 (% w/v) ficoll 400 as crowding agent.

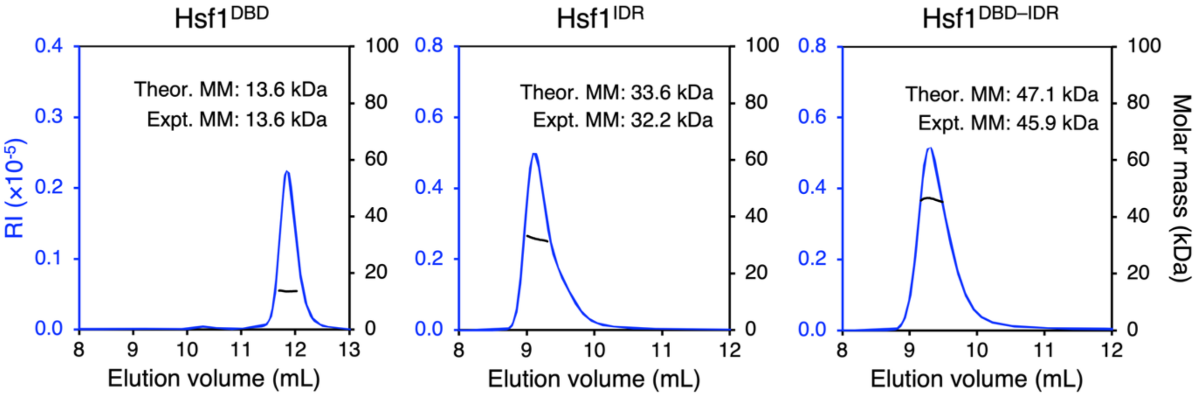

Figure S2. SEC–MALS of Hsf1 constructs. SEC–MALS of Hsf1^{DBD} (left panel), Hsf1^{IDR} (center panel), and Hsf1$^{DBD–IDR}$ (right panel).

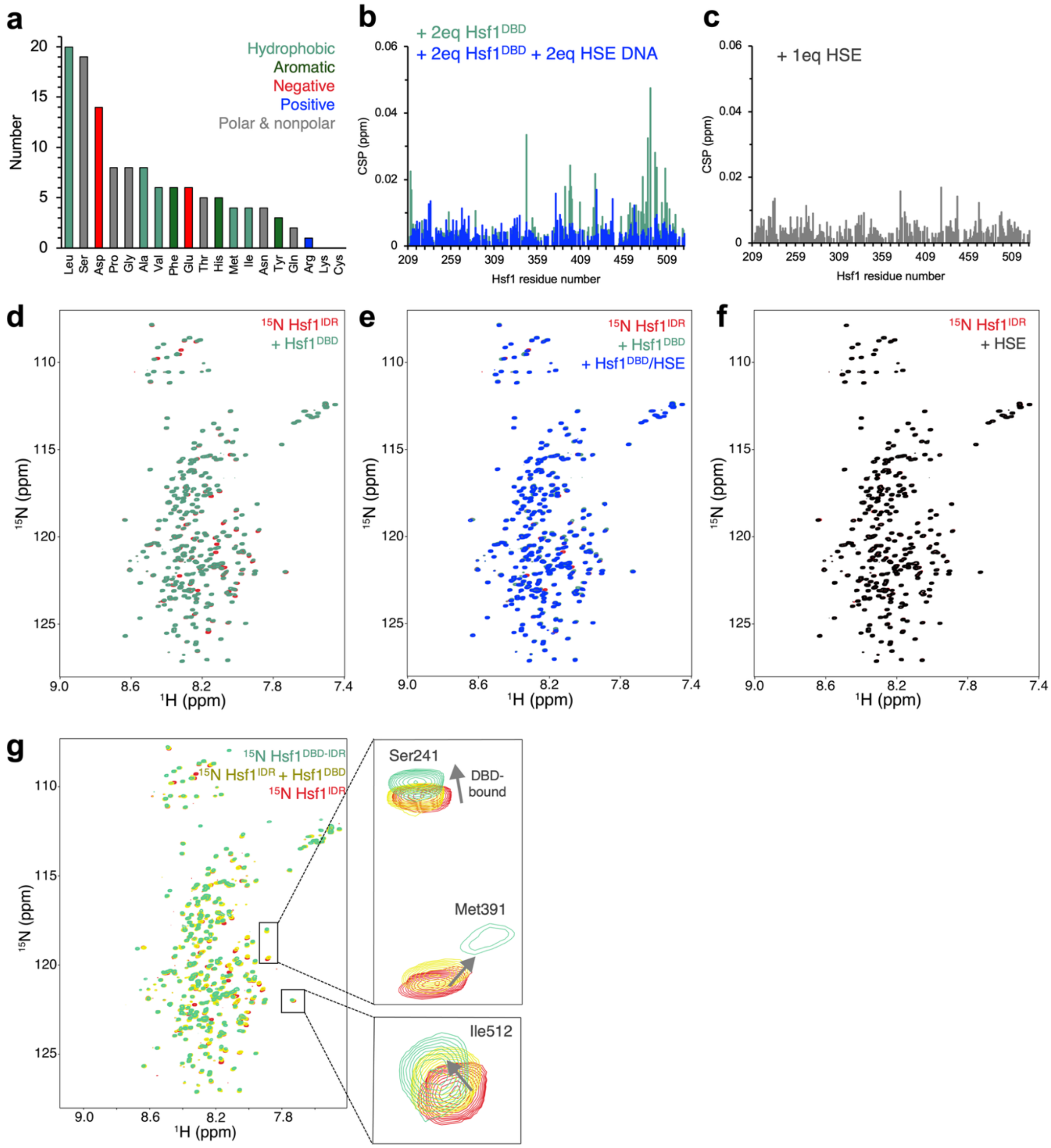

Figure S3. NMR interaction analysis using ^{15}N Hsf1^{IDR}. (a) Residue-type distribution of the DBD-binding sites in IDR residues interacting with the DBD, defined as those showing chemical shift changes above the average value in the ^{1}H–^{15}N HSQC spectra of the Hsf1$^{DBD–IDR}$ and Hsf1^{IDR}. (b) CSPs of Hsf1^{IDR} upon binding to Hsf1^{DBD} (green) and Hsf1^{DBD} and HSE DNA (blue). (c) CSPs of Hsf1^{IDR} upon binding to HSE DNA (gray). (d) ^{1}H–^{15}N HSQC spectra of ^{15}N Hsf1^{IDR} in the absence (red) and presence (green) of 2 eq of unlabeled Hsf1^{DBD}. (e) ^{1}H–^{15}N HSQC spectra of ^{15}N Hsf1^{IDR} in the absence (red) and presence of 2 eq of unlabeled Hsf1^{DBD} (red) or HSE DNA (blue). (f) ^{1}H–^{15}N HSQC spectra of ^{15}N Hsf1^{IDR} in the absence (red) and presence of 1 eq of HSE DNA (black). (g) ^{1}H–^{15}N HSQC spectra of ^{15}N Hsf1^{IDR} in the absence (red) and presence (yellow) of 2 eq of unlabeled Hsf1^{DBD} and ^{15}N Hsf1$^{DBD-IDR}$.

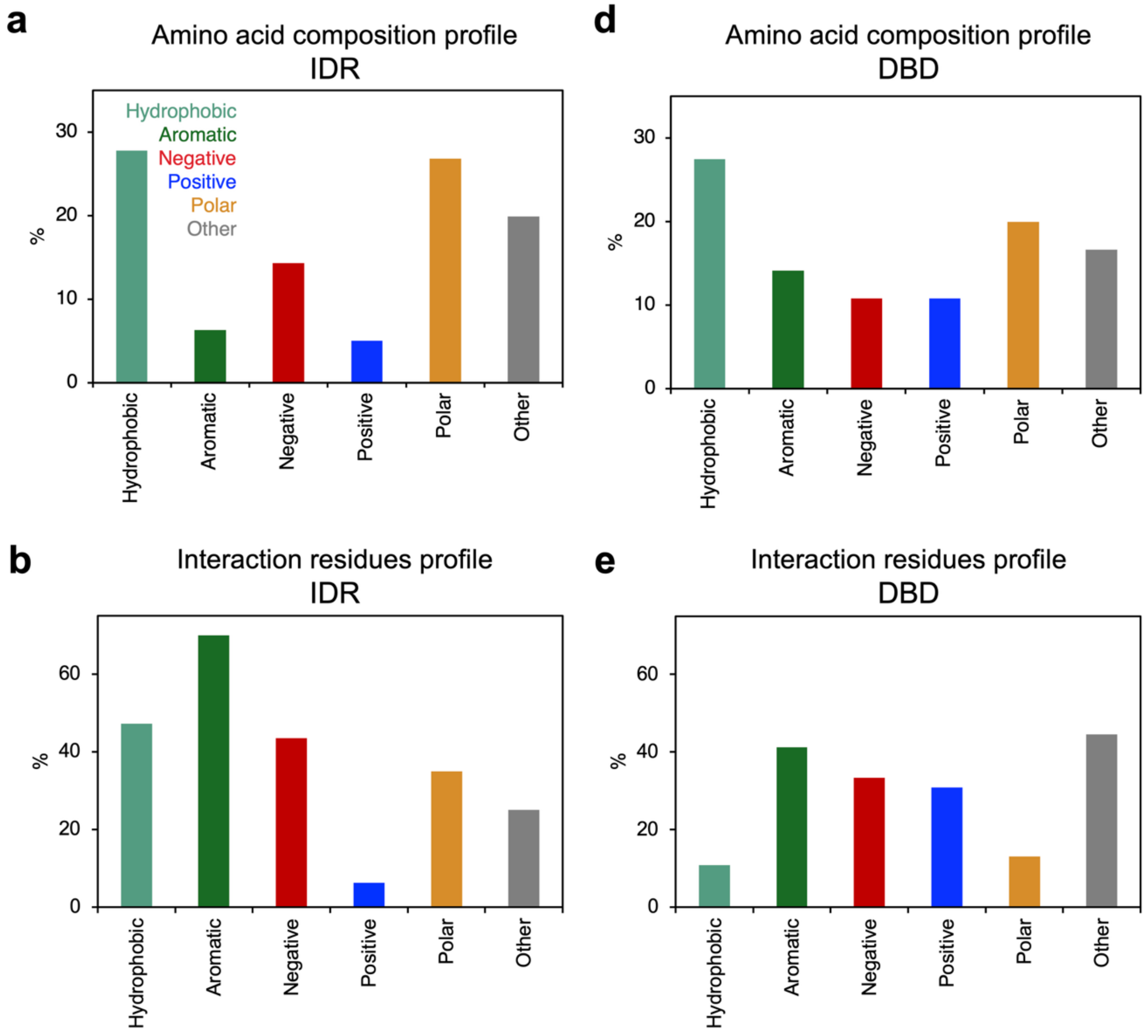

c DBD interaction sites on the IDR

```
209          IP LMLNDSGSAH SMPKYSRQFS LEHVHGSGPY SAPSPAYSSS
251 SLYAPDAVAS SGPIISDITE LAPASPMASP GGSIDERPLS SSPLVRVKEE
301 PPSPPQSPRV EEASPGRPSS VDTLLSPTAL IDSILRESEP APASVTALTD
351 ARGHTDTEGR PPSPPPTSTP EKCLSVACLD KNELSDHLDA MDSNLDNLQT
401 MLSSHGFSVD TSALLDLFSP SVTVPDMSLP DLDSSLASIQ ELLSPQEPPR
451 PPEAENSSPD SGKQLVHYTA QPLFLLDPGS VDTGSNDLPV LFELGEGSYF
501 SEGDGFAEDP TISLLTGSEP PKAKDPTVS
```

Figure S4. Amino acid composition profile of the Hsf1. The percentage values of the amino acid categories within the $Hsf1^{IDR}$ sequence (a) and $Hsf1^{DBD}$ sequence (d). Percentages of the residues in each amino acid category that are included in the interaction sites within the $Hsf1^{IDR}$ sequence (b) and the $Hsf1^{DBD}$ sequence (e). Amino acid categories: Hydrophobic (ILVMA), aromatic (FHWY), negatively charged (DE), positively charged (KR), polar (NQST), and other residues (CGP). (c) Primary sequence of $Hsf1^{IDR}$ with the $Hsf1^{DBD}$-interaction sites highlighted.

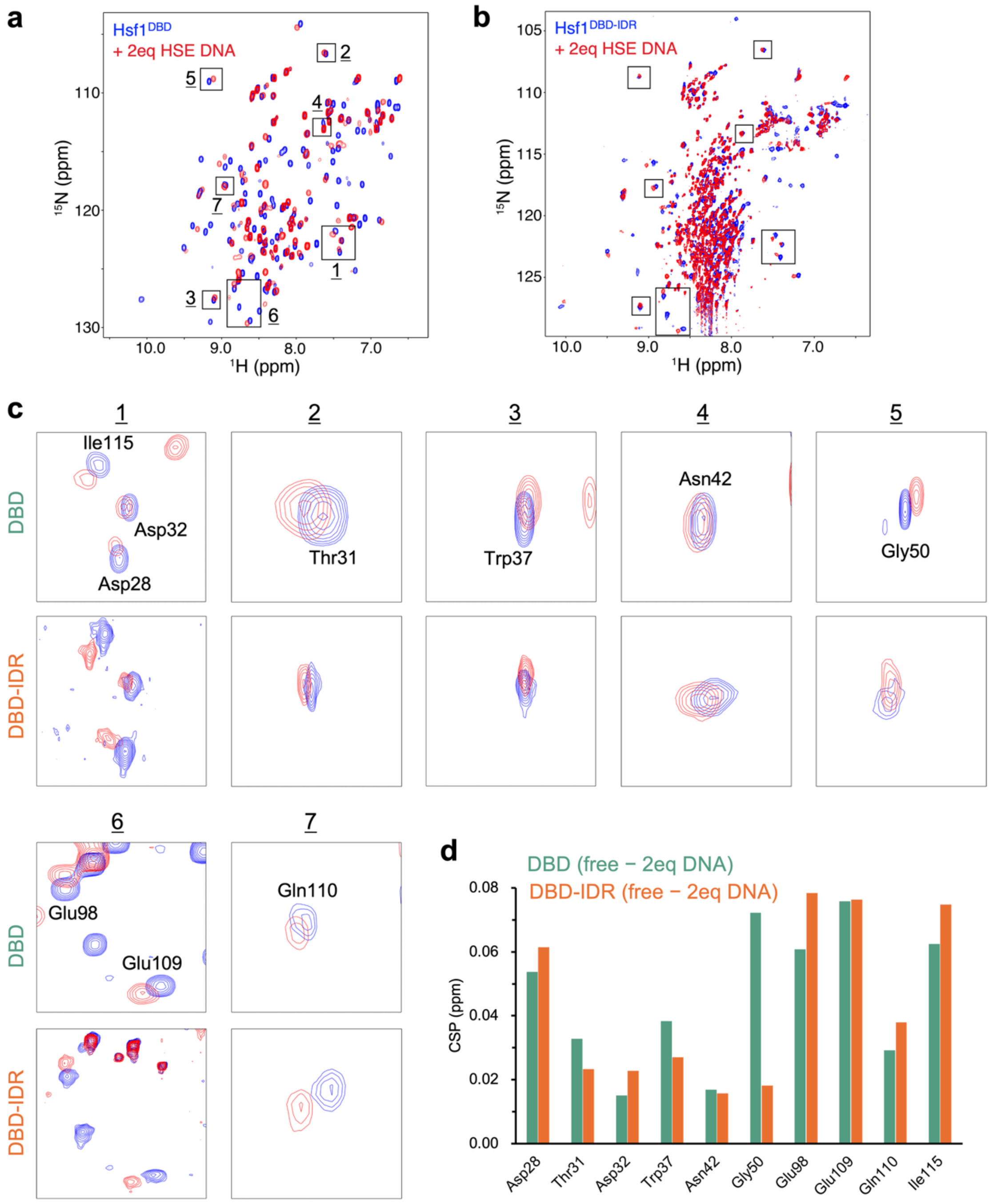

Figure S5. NMR analysis of the interactions between HSE DNA and Hsf1^{DBD} or Hsf1$^{DBD\text{-}IDR}$. (a) ^{1}H–^{15}N HSQC spectra of Hsf1^{DBD} in the absence (blue) and presence of 2.0 eq of HSE DNA (red). (b) ^{1}H–^{15}N HSQC spectra of Hsf1$^{DBD\text{-}IDR}$ in the absence (blue) and presence of 2.0 eq of HSE DNA (red). (c) Selected regions (boxes 1–7) are shown as expanded views. (d) Backbone CSP plots of Hsf1^{DBD} (green) or Hsf1$^{DBD\text{-}IDR}$ (orange) upon the addition of 2 eq HSE DNA. The comparison of the DNA-induced perturbations between Hsf1^{DBD} and Hsf1$^{DBD\text{-}IDR}$ showed no significant difference, indicating that the disordered tail does not substantially affect the binding affinity between the DBD and HSE DNA.

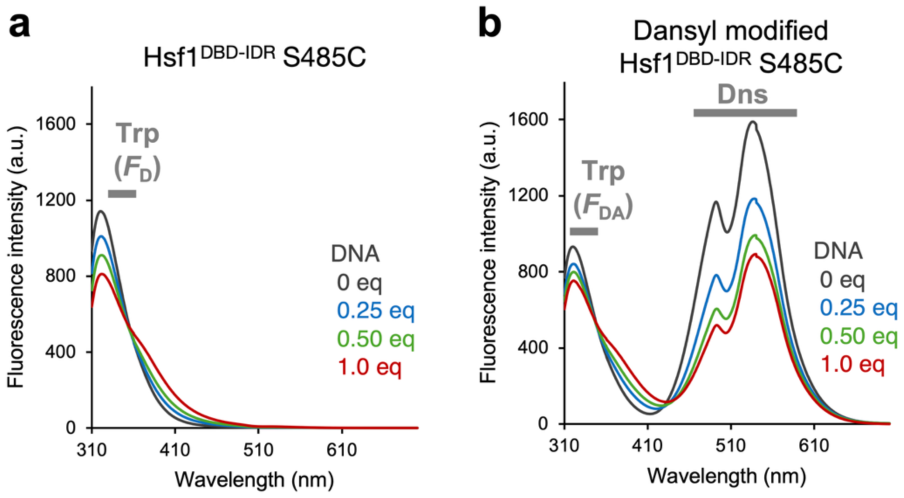

Figure S6. Evaluation of structural changes upon DNA binding to the DBD–IDR using a FRET assay. (a, b) Fluorescence spectra before (a) and after (b) dansyl modification, with excitation at 295 nm corresponding to Trp absorption. After dansyl modification, Trp-dansyl FRET was observed.

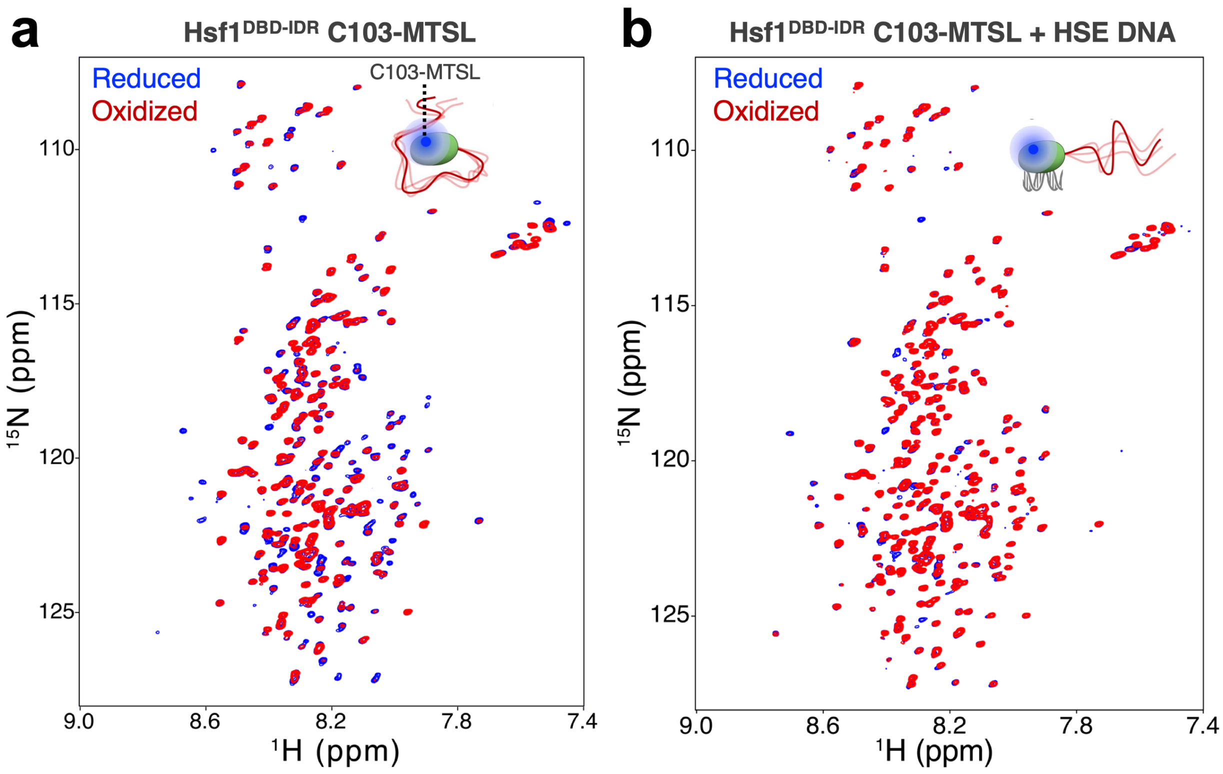

Figure S7. PRE experiments using Hsf1$^{DBD–IDR}$ labeled with C103-MTSL. (a, b) $^{1}H–^{15}N$ HSQC spectra of Hsf1$^{DBD–IDR}$ labeled with C103-MTSL in reduced condition with ascorbic acid (blue) and oxidized condition (red) in the absence (a) and presence of 2 eq HSE DNA (b).

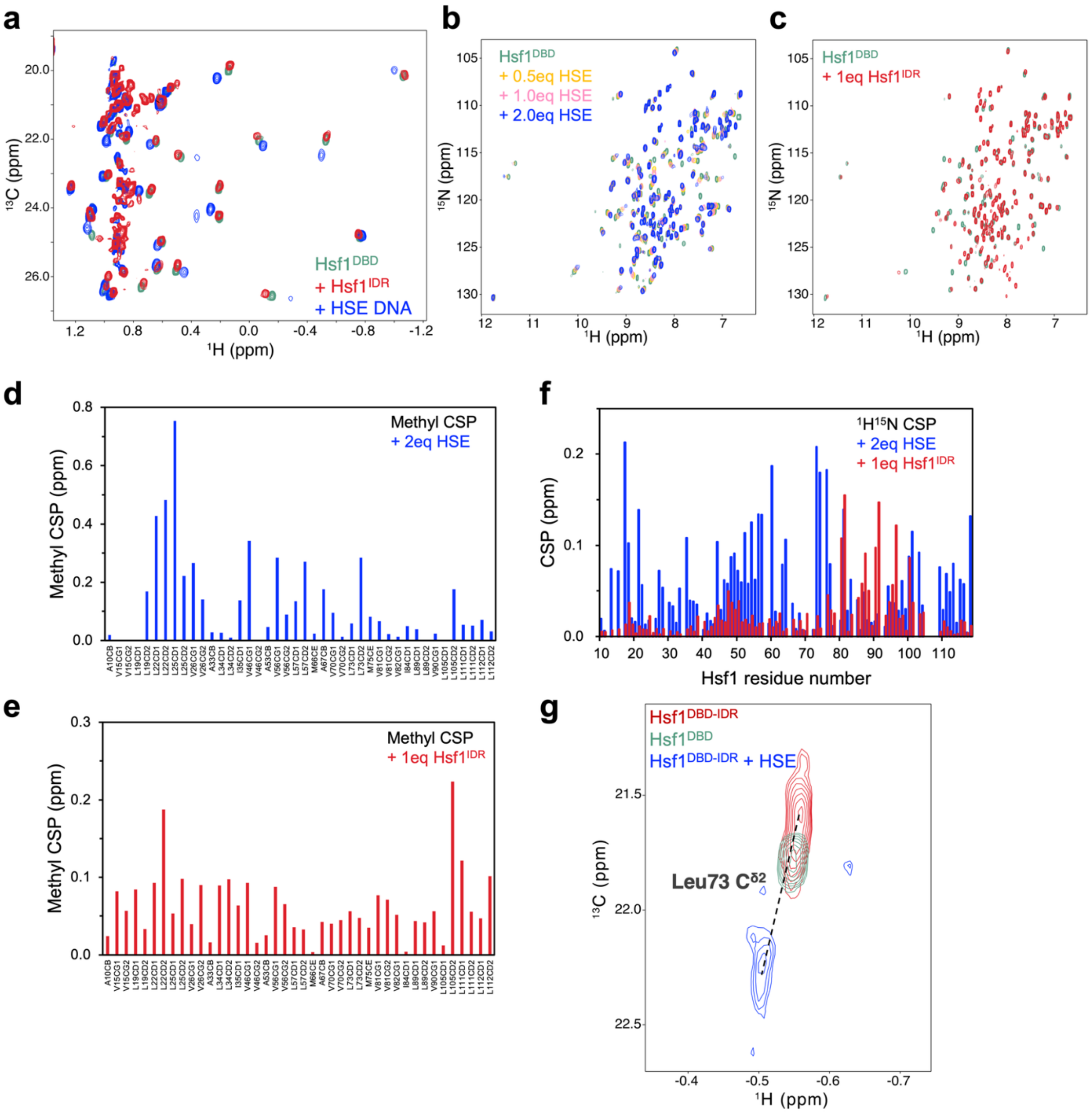

Figure S8. Chemical shift perturbations between free and ligand-bound states of Hsf1^{DBD}. (a) ^{1}H–^{13}C HMQC spectra of Hsf1^{DBD} in the absence (green) and presence of 2 eq HSE DNA (blue) or 2 eq Hsf1^{IDR} (red). (b) ^{1}H–^{15}N HSQC spectra of Hsf1^{DBD} in the absence (green) and presence of 0.5 eq (yellow), 1.0 eq (pink), and 2.0 eq (blue) of HSE DNA. (c) ^{1}H–^{15}N HSQC spectra of Hsf1^{DBD} in the absence (green) and presence of 2 eq Hsf1^{IDR} (red). (d, e) Methyl CSP plots of Hsf1^{DBD} upon binding to 2 eq HSE DNA (d) and 2 eq Hsf1^{IDR} (e). (f) Backbone CSP plots of Hsf1^{DBD} upon binding to 2 eq HSE DNA (blue) and 2 eq Hsf1^{IDR} (red). (g) Overlaid ^{1}H–^{13}C HMQC spectra of the free Hsf1^{DBD} (green), free Hsf1$^{DBD\text{-}IDR}$ (red), and DNA-bound Hsf1$^{DBD\text{-}IDR}$ (blue).

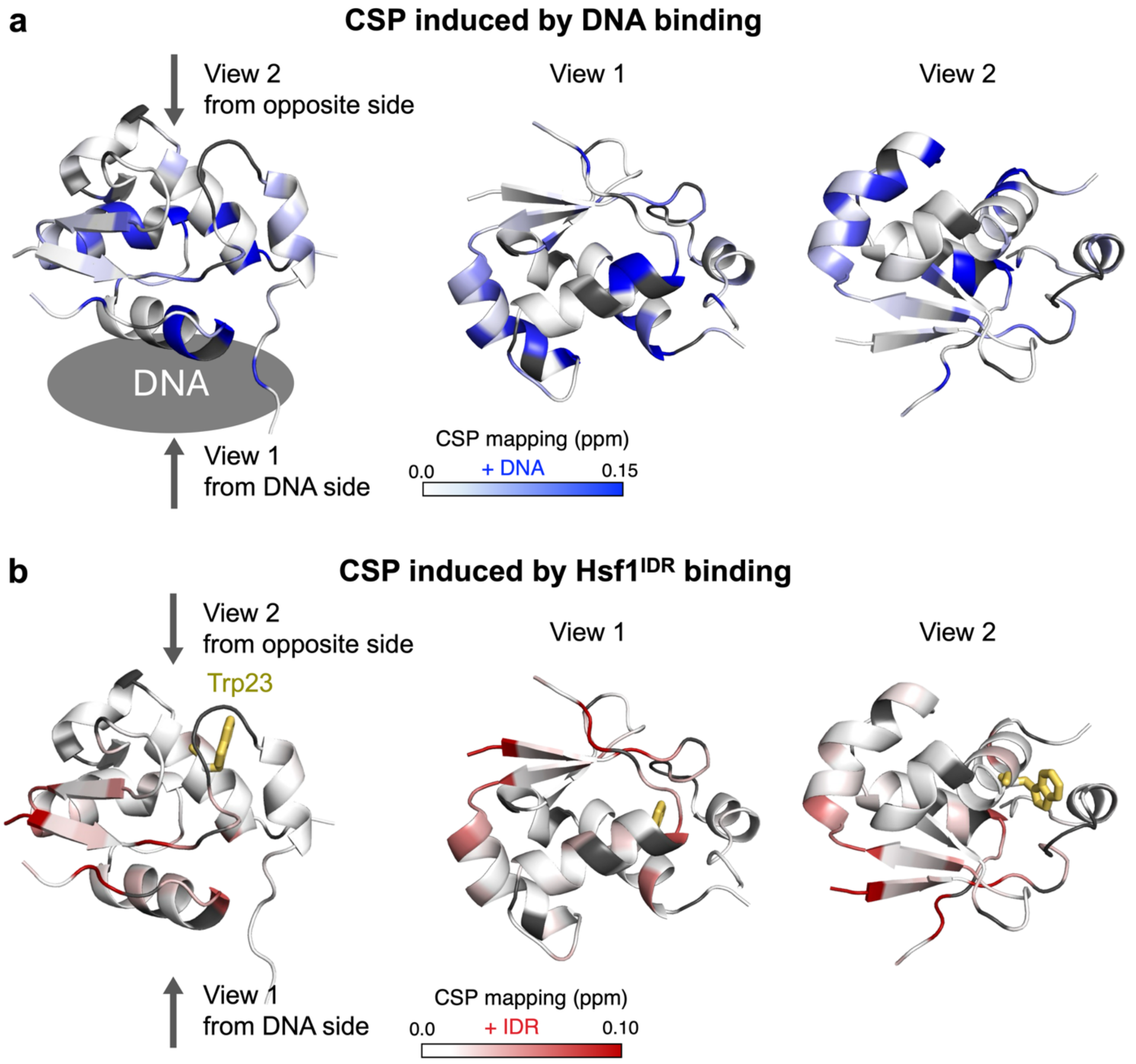

Figure S9. CSP mapping on Hsf1^{DBD} induced by DNA and Hsf1^{IDR} binding. (a) CSP induced by DNA binding were mapped onto the structure of the Hsf1^{DBD} and are shown in blue. Two opposite views are presented. The left panel indicates the approximate position of bound DNA (gray ellipse) and the orientations corresponding to view 1 and view 2. (b) CSPs induced by Hsf1^{IDR} binding were mapped onto the same structure and are shown in red. Trp23 is shown as yellow sticks.

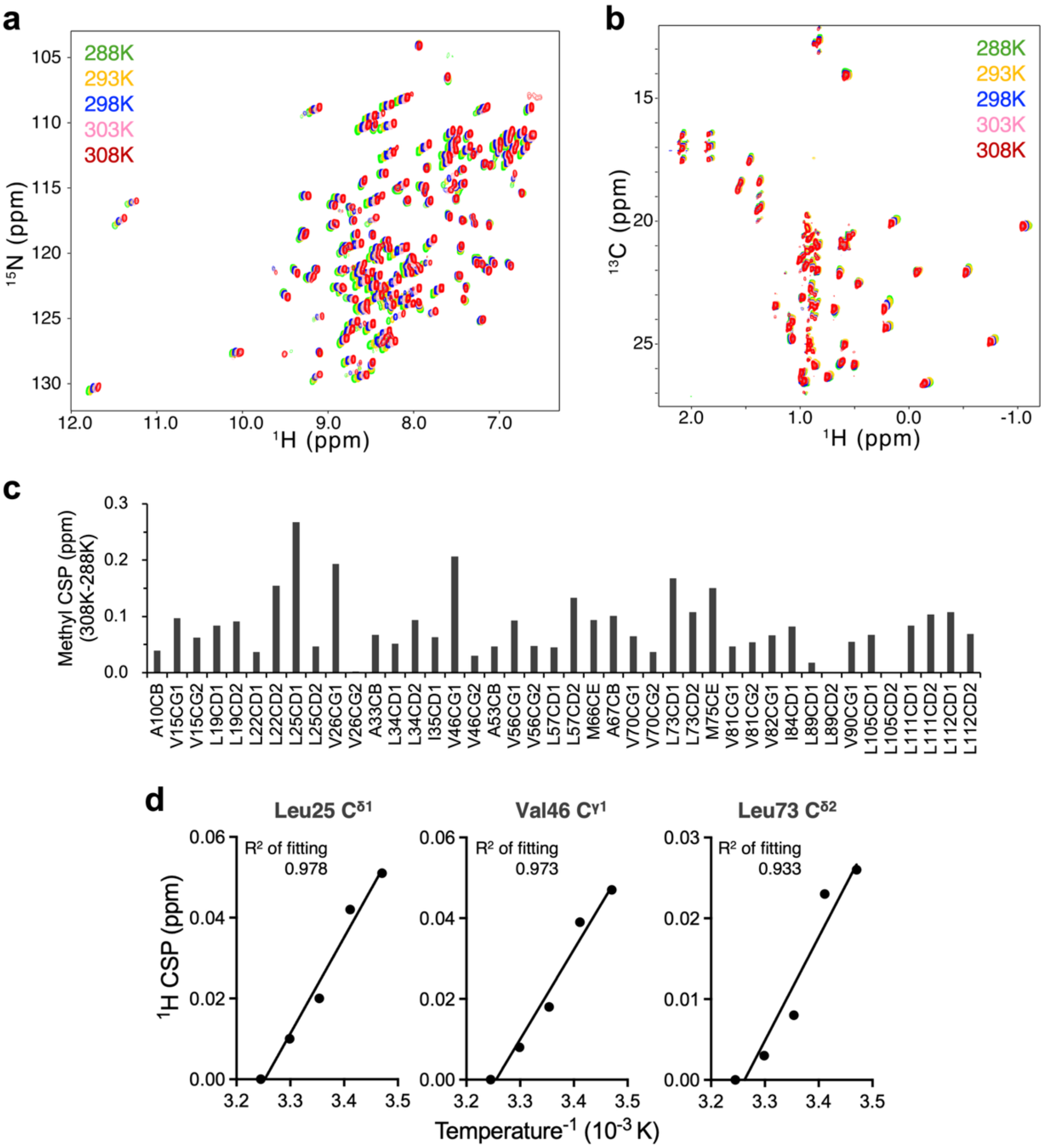

Figure S10. Temperature-dependent changes in the NMR spectra of Hsf1^{DBD}. (a, b) Temperature dependence of the backbone amide signals (a) and the methyl signals (b) of free Hsf1^{DBD}. (c) Methyl CSP plots of Hsf1^{DBD} in response to the temperature change from 288 K to 308 K. (d) Van't Hoff plot of three resonances that showed the large temperature-dependent ^{1}H chemical shift changes from methyl signals.

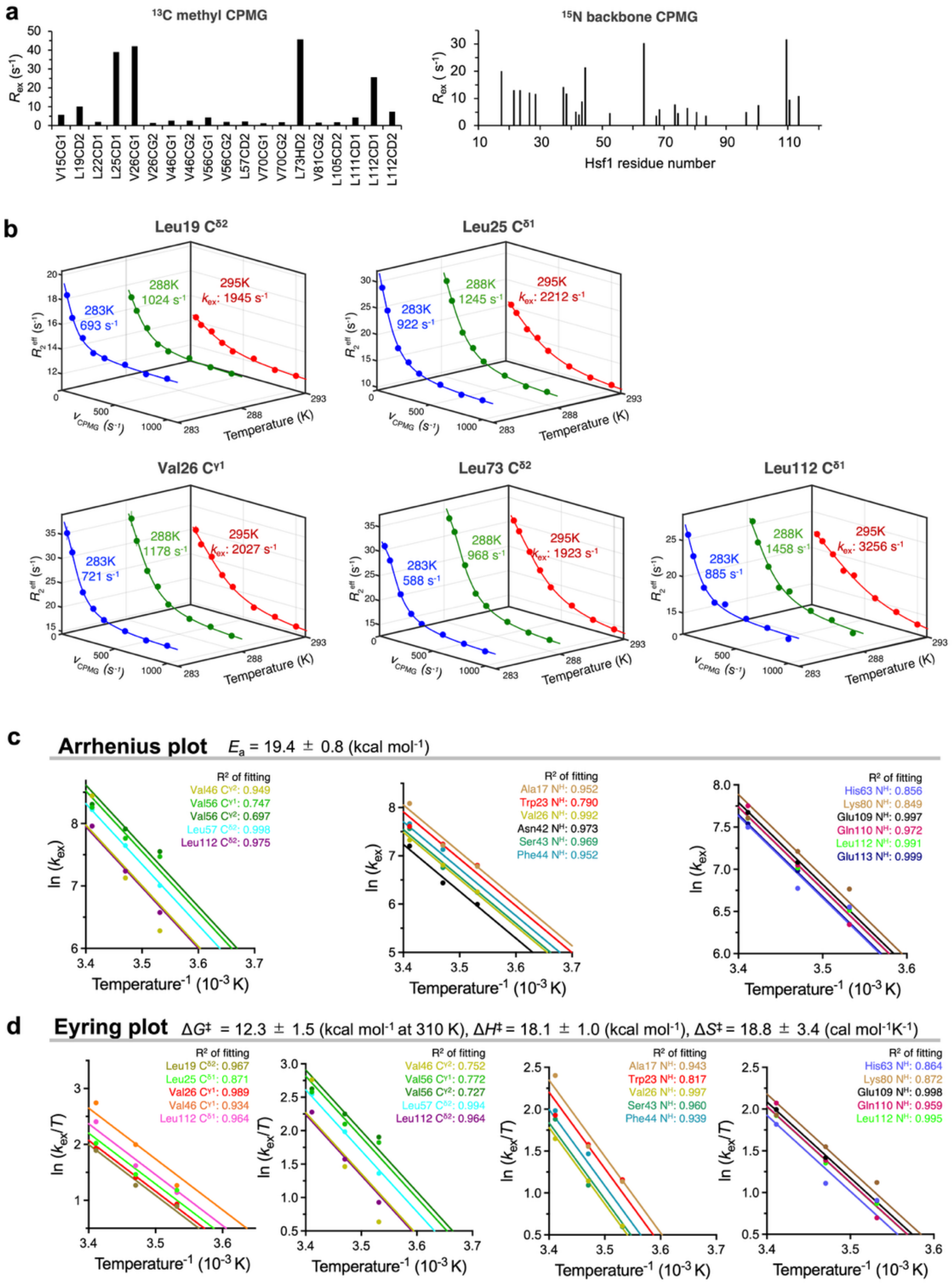

Figure S11. Relaxation dispersion analysis of Hsf1^{DBD} in the free state. (a) R_{ex} values of Hsf1^{DBD} in methyl ^{13}C or backbone ^{15}N CPMG RD experiments at 800 MHz, assuming two-site exchange. (b) R_{ex} values of Hsf1^{DBD} in methyl RD experiments at 800 MHz, assuming two-site exchange. Error bars were derived from the noise level of the NMR spectra. (c) The calculated exchange rates (k_{ex}) of backbone moieties are plotted following the Arrhenius equation at different temperatures. The data are reported only when the amplitudes of their relaxation dispersion profiles are well above the experimental errors. The data are presented in three separate panels for clarity. (d) Eyring plots of ln (k_{ex}/T) versus temperature^{-1} were fitted using the Eyring equation to estimate the apparent activation parameters of the exchange process. The data are presented in four separate panels for clarity.

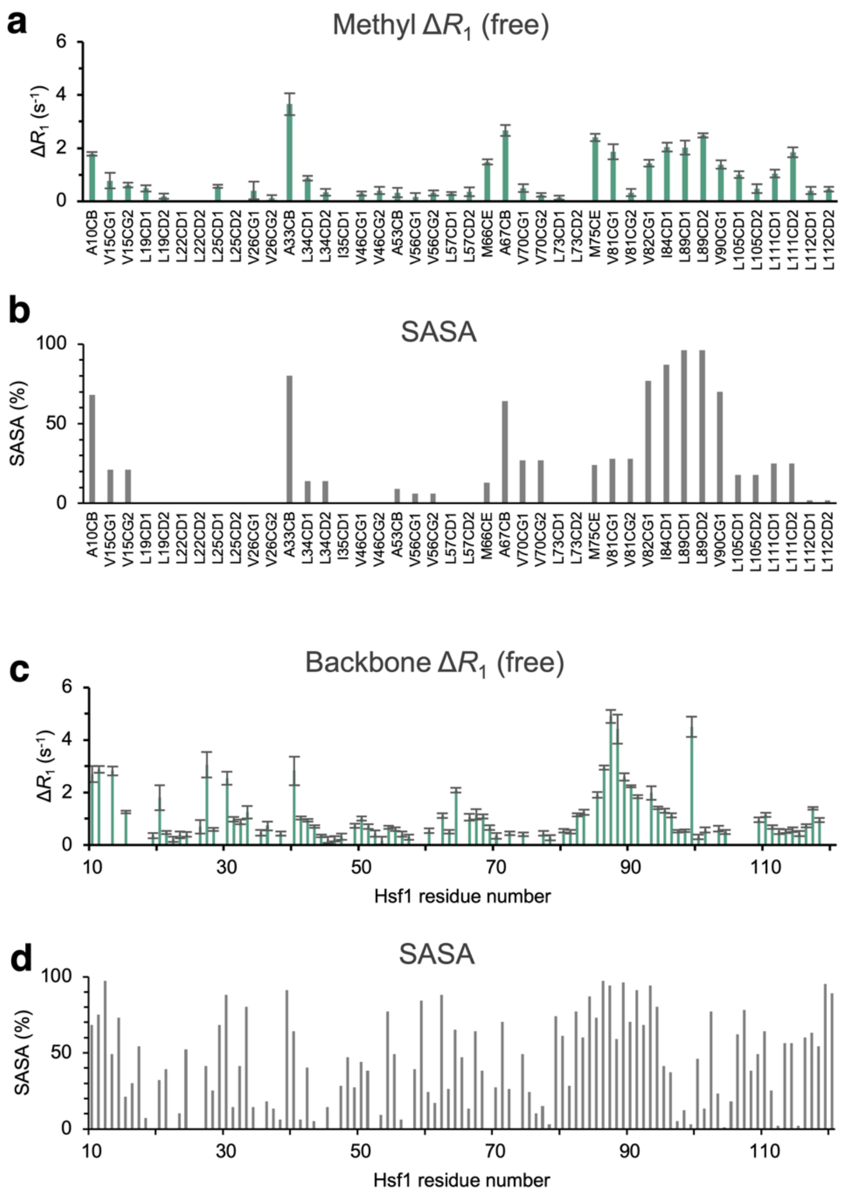

Figure S12. sPREs of the ligand-free Hsf1^{DBD}. (a) sPREs on the longitudinal relaxation rates (ΔR_1) of the methyl resonances of Hsf1^{DBD} upon addition of 2 mM Gadodiamide. Error bars were calculated from the fitting errors obtained when determining R_1 values before and after the addition of Gadodiamide. (b) The SASA of the DBD methyl group calculated using PyMOL. (c) ΔR_1 of the backbone signals of Hsf1^{DBD} upon addition of 2 mM Gadodiamide. Error bars were calculated from the fitting errors obtained when determining R_1 values before and after the addition of Gadodiamide. (d) The SASA of the DBD backbone calculated using PyMOL.

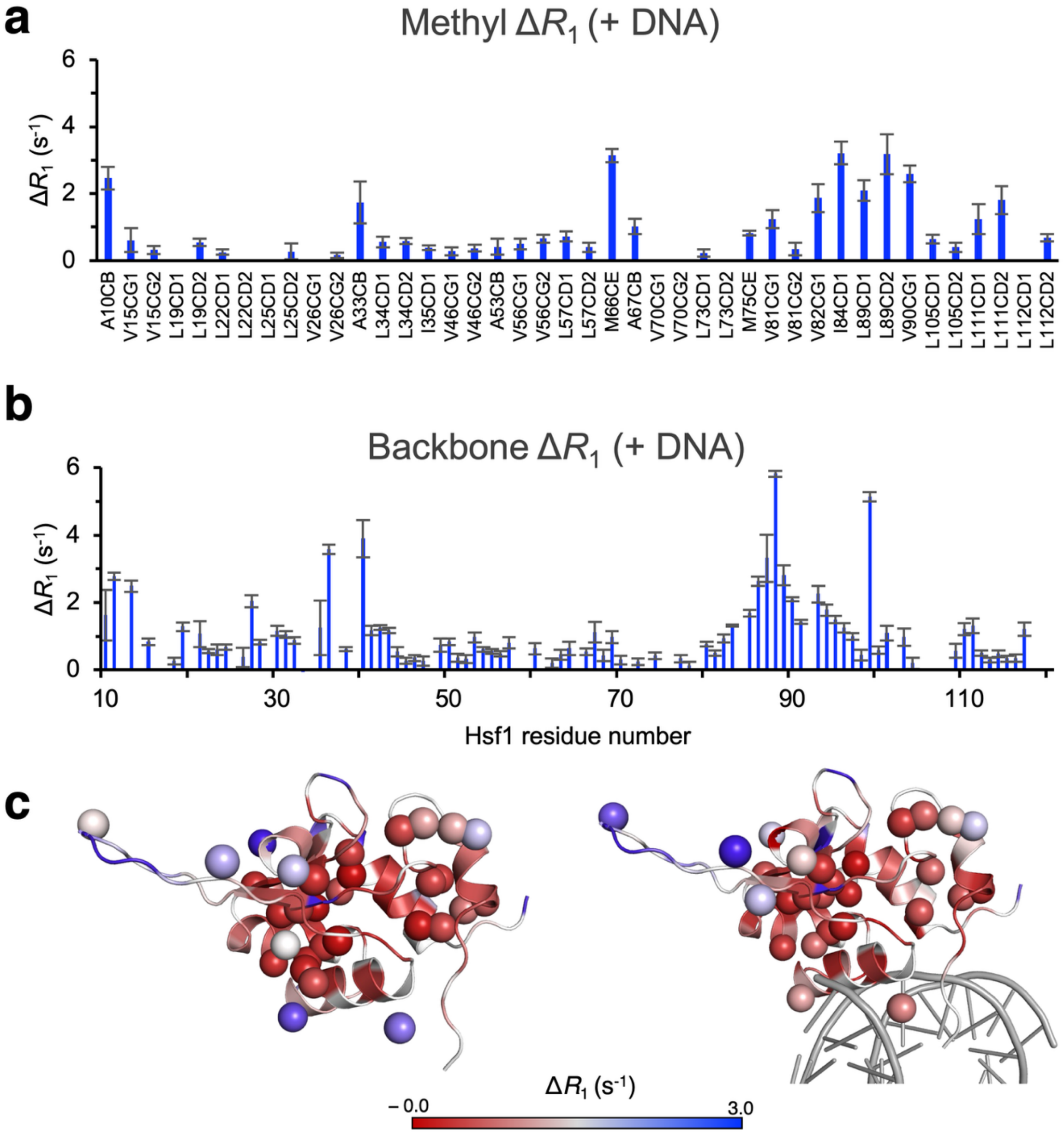

Figure S13. sPREs of Hsf1^{DBD} with HSE DNA. (a) ΔR_1 of the methyl resonances of Hsf1^{DBD} upon addition of 2 mM Gadodiamide in the presence of HSE DNA. Error bars were calculated from the fitting errors obtained when determining R_1 values before and after the addition of Gadodiamide. (b) ΔR_1 of the backbone signals of Hsf1^{DBD} upon addition of 2 mM Gadodiamide in the presence of HSE DNA. Error bars were calculated from the fitting errors obtained when determining R_1 values before and after the addition of Gadodiamide. (c) Mapping of ΔR_1 onto the DBD structure for the ligand-free state (left) and DNA-bound state (right), respectively.

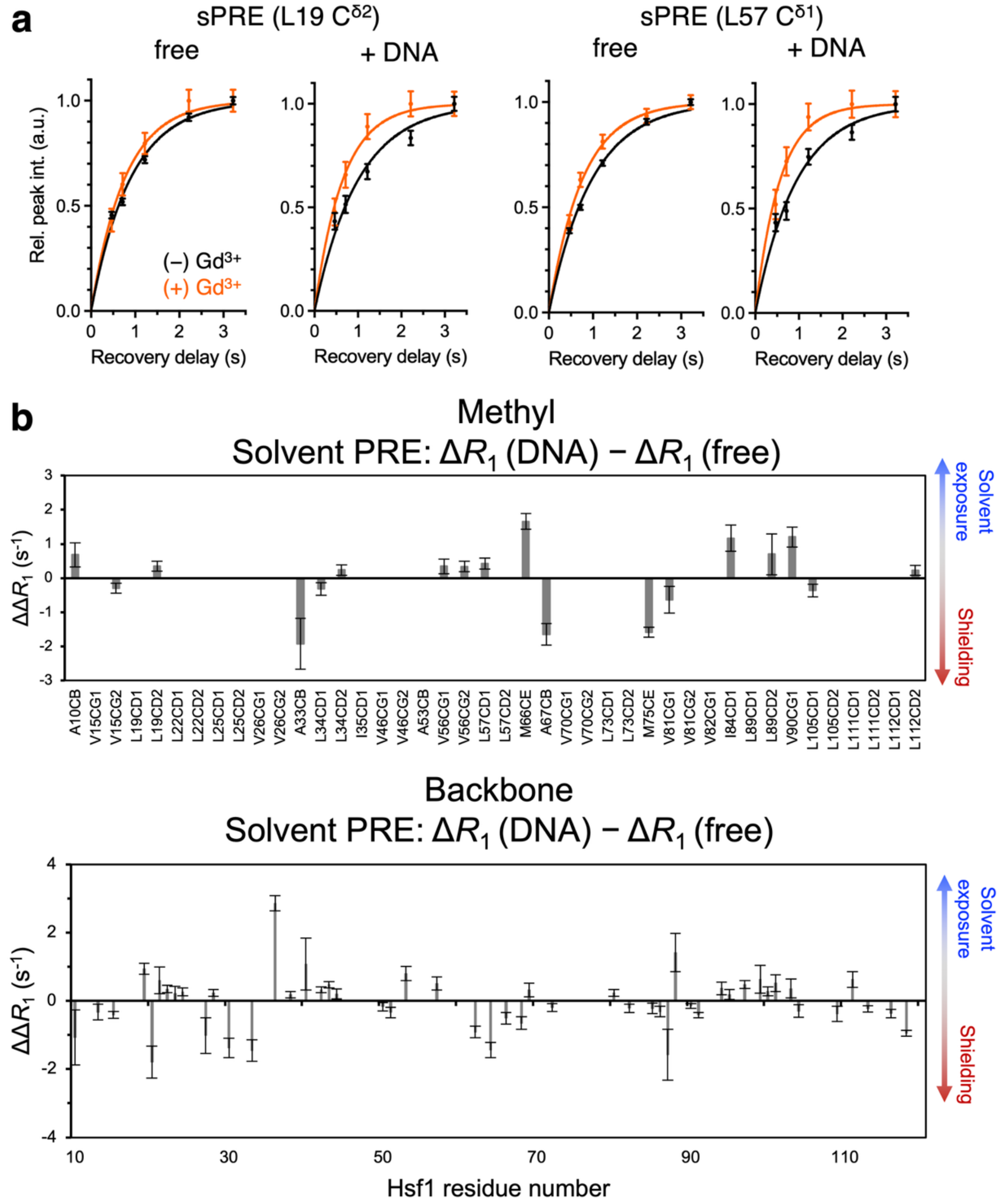

Figure S14. sPRE changes of Hsf1^{DBD} induced by DNA binding. (a) Measurements of the R_1 relaxation rate in the presence and absence of 2 mM gadodiamide. Normalized signal intensities are plotted against recovery delay. Error bars were derived from the noise level of the NMR spectra. (b) Effect of DNA binding on ΔR_1 of methyl (top panel) and backbone (bottom panel) signals of Hsf1^{DBD} shown as $\Delta\Delta R_1$. Positive values indicate increased solvent exposure upon DNA binding, while negative values indicate protection from solvent. Error bars represent fitting errors from exponential curve fitting.

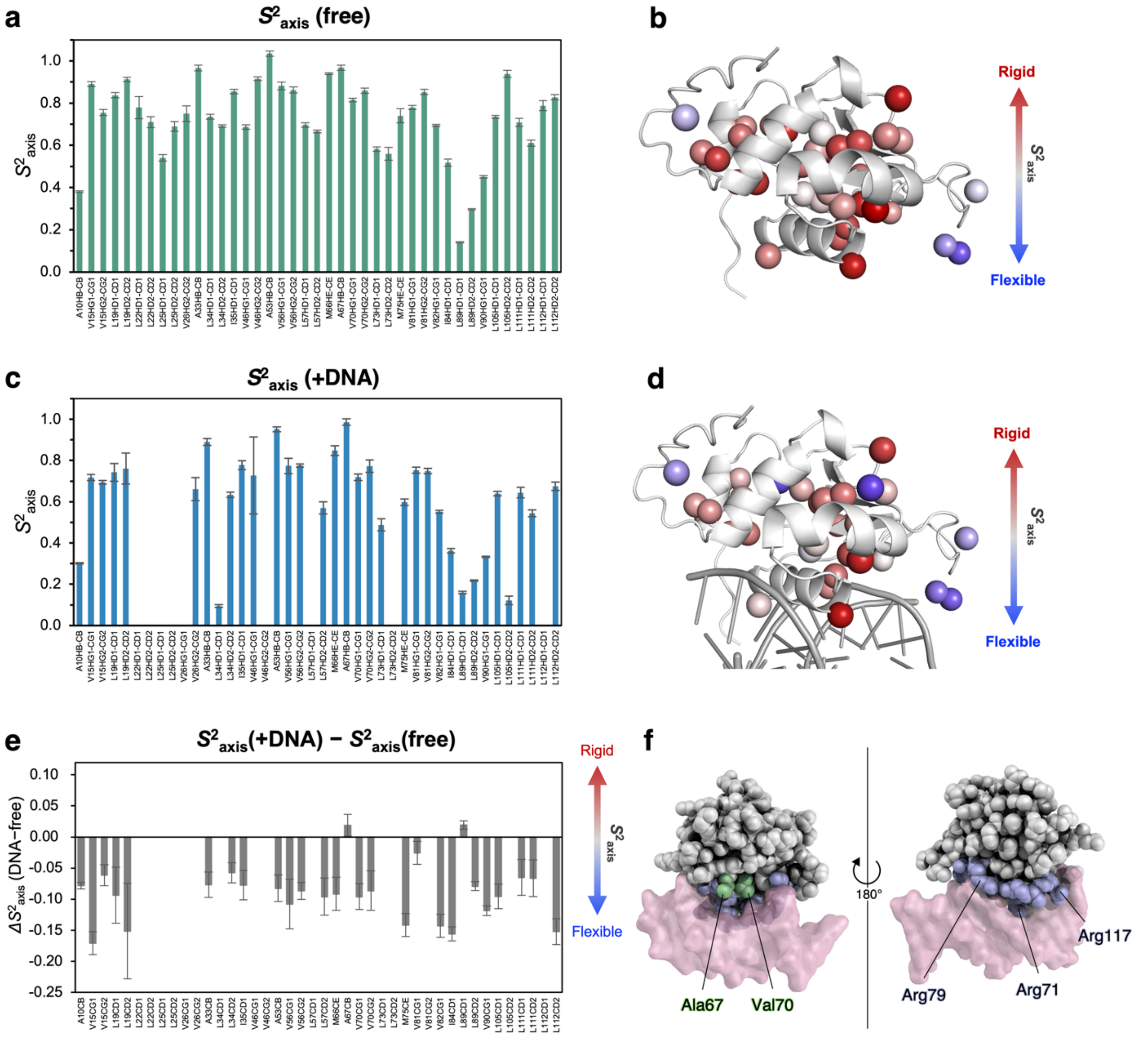

Figure S15. Side-chain dynamics of Hsf1^{DBD} in the free and DNA-bound states. (a, b) S^2_{axis} of the methyl groups of Hsf1^{DBD} in the free state (a) and their structural mapping (b). Residues with high S^2_{axis} are rigid (red), while those with low S^2_{axis} are flexible (blue). Error bars represent fitting errors from curve fitting. (c, d) S^2_{axis} of methyl groups in the DNA-bound state (c) and their structural mapping (d). (e) Effect of DNA binding on the methyl order parameters, S^2_{axis}, of Hsf1^{DBD}. Bar graphs showing changes in methyl group order parameters (ΔS^2axis). (f) Specific interactions of Hsf1^{DBD} with the DNA (PDBID: 5D5V). Hsf1^{DBD} is shown as gray spheres, and the DNA as a pink surface. Among the residues in the vicinity of the DNA-binding interface, methyl-containing residues are shown in green and charged residues in blue. Charged residues such as Arg79, Arg71, and Arg117 directly contact with DNA, whereas methyl-containing residues such as Ala67 and Val70 have no major contact with DNA and remain exposed on the surface.

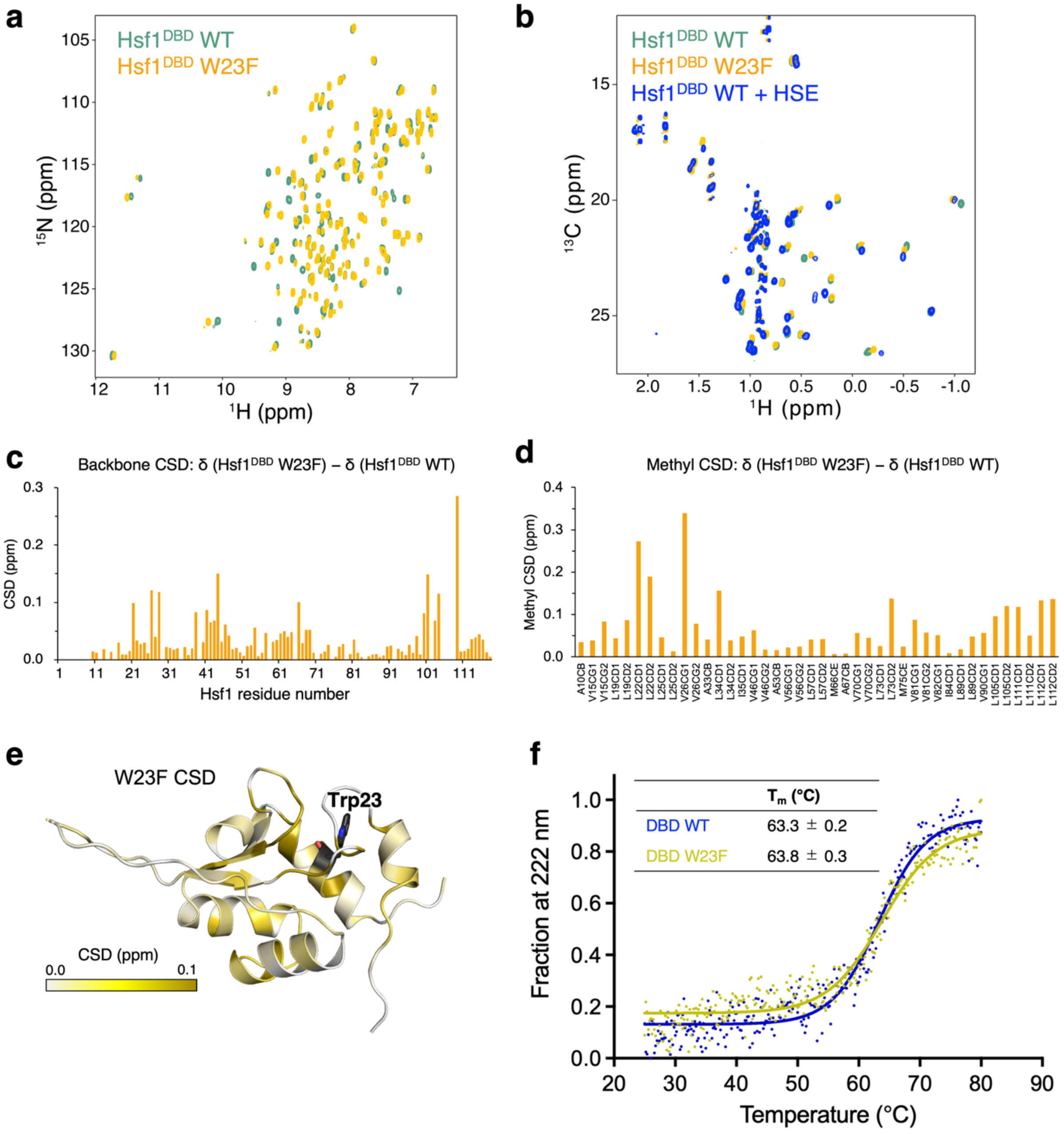

Figure S16. Assessment of the effect of mutations at Trp23 on the structure of Hsf1^{DBD}. (a) ^{1}H–^{15}N HSQC spectra of Hsf1^{DBD} WT (green) and W23F (yellow). (b) ^{1}H–^{13}C HMQC spectra of Hsf1^{DBD} WT (green), W23F (yellow), and Hsf1^{DBD} WT with 2eq HSE DNA (blue). (c) Backbone CSD plots of the DBD by W23F mutation. (d) Methyl CSD plots of the DBD by W23F mutation. (e) Mapping of residues that showed substantial CSD on backbone signals caused by the W23F mutation. (f) Thermal denaturation profiles of Hsf1^{DBD} WT and W23F monitored by circular dichroism at 222 nm. Solid lines indicate fitted curves, and the corresponding melting temperatures (T_m) are indicated in the inset.

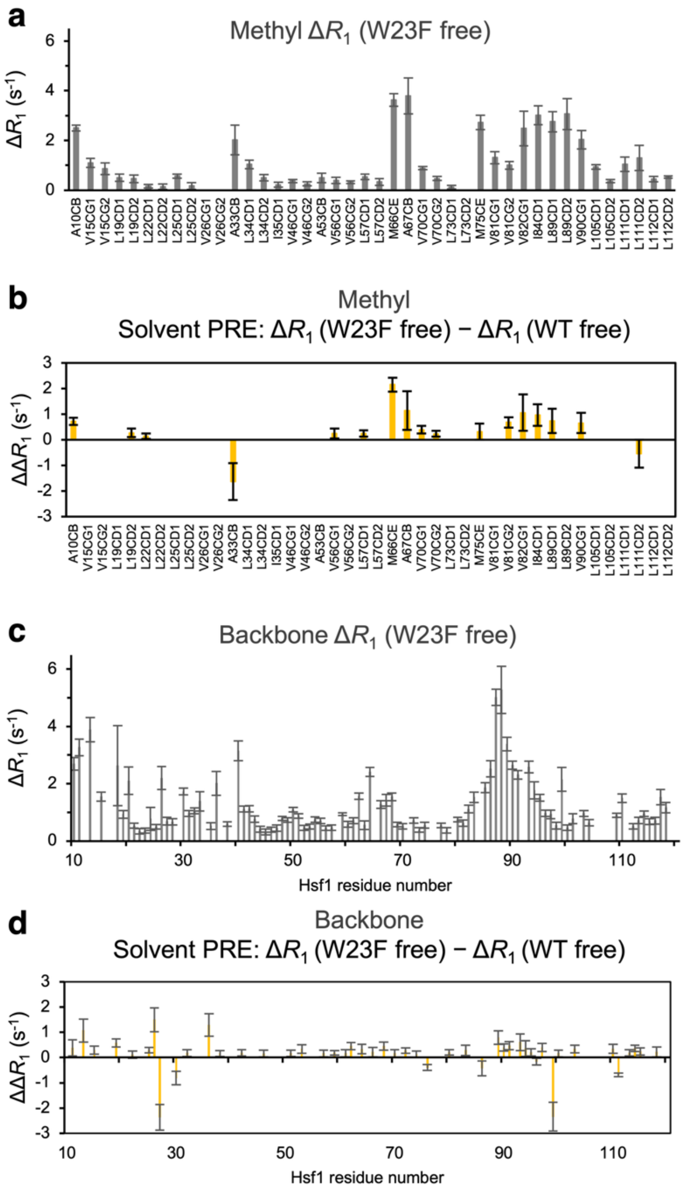

Figure S17. sPRE of the free DBD W23F mutant. (a) Solvent PREs upon addition of 2 mM Gadodiamide. Error bars were calculated from the fitting errors obtained when determining R_1 values before and after the addition of Gadodiamide, respectively. (b) Effect of W23F mutation towards methyl signals of Hsf1^{DBD}, as represented by the sPRE difference $\Delta\Delta R_1$. Positive values indicate increased solvent exposure upon DNA binding, while negative values indicate protection from solvent. (c) Solvent PREs upon addition of 2 mM Gadodiamide. (d) Effect of W23F mutation towards backbone signals on solvent PRE effects shown as $\Delta\Delta R_1$.

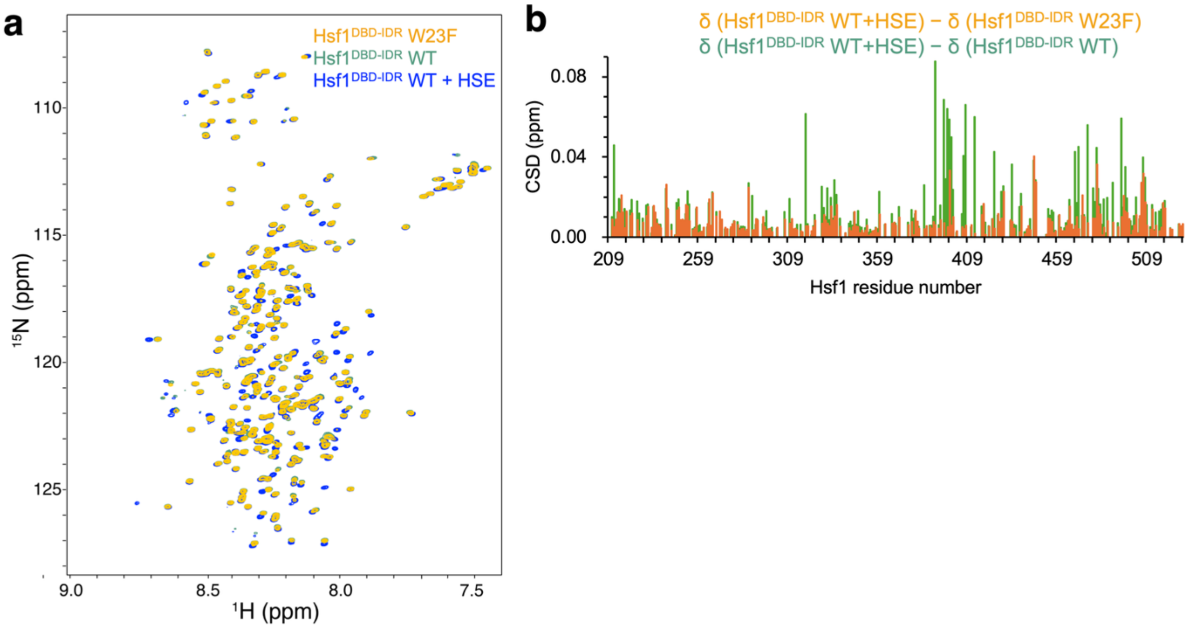

Figure S18. Allosteric W23F mutation affects the DBD–IDR interaction. (a) ^{1}H–^{15}N HSQC spectra of free Hsf1$^{DBD–IDR}$ (green), in complex with HSE DNA (blue), and free Hsf1$^{DBD-IDR}$ W23F (yellow). (b) CSDs from Hsf1$^{DBD–IDR}$ in complex with DNA to Hsf1$^{DBD-IDR}$ (green), and from Hsf1$^{DBD–IDR}$ in complex with DNA to Hsf1$^{DBD–IDR}$ W23F (yellow).

References

[1] F. Delaglio, S. Grzesiek, G. W. Vuister, G. Zhu, J. Pfeifer, A. Bax, “NMRPipe: A multidimensional spectral processing system based on UNIX pipes” *J Biomol NMR* **1995**, *6*, 277–293.

[2] J. P. Loria, M. Rance, A. G. Palmer, “A TROSY CPMG sequence for characterizing chemical exchange in large proteins” *J Biomol NMR* **1999**, *15*, 151–155.

[3] V. Tugarinov, L. E. Kay, “Separating Degenerate 1H Transitions in Methyl Group Probes for Single-Quantum 1H-CPMG Relaxation Dispersion NMR Spectroscopy” *J. Am. Chem. Soc.* **2007**, *129*, 9514–9521.

[4] P. Lundström, P. Vallurupalli, T. L. Religa, F. W. Dahlquist, L. E. Kay, “A single-quantum methyl 13C-relaxation dispersion experiment with improved sensitivity” *J Biomol NMR* **2007**, *38*, 79–88.

[5] I. R. Kleckner, M. P. Foster, “GUARDD: user-friendly MATLAB software for rigorous analysis of CPMG RD NMR data” *J Biomol NMR* **2012**, *52*, 11–22.

[6] D. M. Korzhnev, K. Kloiber, L. E. Kay, “Multiple-Quantum Relaxation Dispersion NMR Spectroscopy Probing Millisecond Time-Scale Dynamics in Proteins: Theory and Application” *J. Am. Chem. Soc.* **2004**, *126*, 7320–7329.

[7] H. Eyring, “The Activated Complex in Chemical Reactions” *J. Chem. Phys.* **1935**, *3*, 107–115.

[8] H. Sun, L. E. Kay, V. Tugarinov, “An Optimized Relaxation-Based Coherence Transfer NMR Experiment for the Measurement of Side-Chain Order in Methyl-Protonated, Highly Deuterated Proteins” *J. Phys. Chem. B* **2011**, *115*, 14878–14884.

[9] V. Tugarinov, R. Sprangers, L. E. Kay, “Probing Side-Chain Dynamics in the Proteasome by Relaxation Violated Coherence Transfer NMR Spectroscopy” *J. Am. Chem. Soc.* **2007**, *129*, 1743–1750.

[10] A. Ortega, D. Amorós, J. García de la Torre, “Prediction of Hydrodynamic and Other Solution Properties of Rigid Proteins from Atomic- and Residue-Level Models” *Biophysical Journal* **2011**, *101*, 892–898.

[11] N. J. Greenfield, “Analysis of the kinetics of folding of proteins and peptides using circular dichroism” *Nat Protoc* **2006**, *1*, 2891–2899.
